# Regulatory T cell adoptive transfer alters uterine immune populations, increasing a novel MHC-II^low^ macrophage associated with healthy pregnancy

**DOI:** 10.3389/fimmu.2023.1256453

**Published:** 2023-10-13

**Authors:** Emma L. Lewis, Erin R. Reichenberger, Lauren Anton, Michael V. Gonzalez, Deanne M. Taylor, Paige M. Porrett, Michal A. Elovitz

**Affiliations:** ^1^Center for Research on Reproduction and Women’s Health, Perelman School of Medicine, University of Pennsylvania, Philadelphia, PA, United States; ^2^Department of Biomedical and Health Informatics, Children’s Hospital of Philadelphia, Philadelphia, PA, United States; ^3^Center for Cytokine Storm Treatment & Laboratory, Perelman School of Medicine, University of Pennsylvania, Philadelphia, PA, United States; ^4^Center for Applied Genomics, Children’s Hospital of Philadelphia, Philadelphia, PA, United States; ^5^Department of Pediatrics, Perelman School of Medicine, University of Pennsylvania, Philadelphia, PA, United States; ^6^Department of Surgery, Hospital of the University of Pennsylvania, Philadelphia, PA, United States; ^7^Women’s Biomedical Research Institute, Department of Obstetrics, Gynecology, and Reproductive Sciences, Icahn School of Medicine at Mount Sinai, New York, NY, United States

**Keywords:** reproductive immunology, mucosal immunity, IUFD, single-cell RNA sequencing, immune regulation, regulatory T cells, macrophage

## Abstract

Intrauterine fetal demise (IUFD) – fetal loss after 20 weeks – affects 6 pregnancies per 1,000 live births in the United States, and the majority are of unknown etiology. Maternal systemic regulatory T cell (Treg) deficits have been implicated in fetal loss, but whether mucosal immune cells at the maternal-fetal interface contribute to fetal loss is under-explored. We hypothesized that the immune cell composition and function of the uterine mucosa would contribute to the pathogenesis of IUFD. To investigate local immune mechanisms of IUFD, we used the CBA mouse strain, which naturally has mid-late gestation fetal loss. We performed a Treg adoptive transfer and interrogated both pregnancy outcomes and the impact of systemic maternal Tregs on mucosal immune populations at the maternal-fetal interface. Treg transfer prevented fetal loss and increased an MHC-II^low^ population of uterine macrophages. Single-cell RNA-sequencing was utilized to precisely evaluate the impact of systemic Tregs on uterine myeloid populations. A population of C1q+, Trem2+, MHC-II^low^ uterine macrophages were increased in Treg-recipient mice. The transcriptional signature of this novel uterine macrophage subtype is enriched in multiple studies of human healthy decidual macrophages, suggesting a conserved role for these macrophages in preventing fetal loss.

## Introduction

Human fetal loss after 20 weeks of gestation is known as an intrauterine fetal demise (IUFD), commonly referred to as stillbirth. In the United States, IUFD affects 6 pregnancies for every 1,000 live births (1, 2), and the cause of death in IUFD is unknown in nearly two-thirds of cases (3). The continued high rate of IUFD and lack of data led to a congressional mandate for the formation of a “Stillbirth Task Force” in 2022 (4). These initiatives have improved data collection on IUFD without elucidating the underlying biology (4). Certain features are frequently present in cases of IUFD of unknown etiology – abnormal placentation, intrauterine growth restriction, histological chorioamnionitis, and elevated maternal white blood cell counts (5). Placental lesions and fetal growth restriction are associated with immune abnormalities (6, 7); collectively these features point toward an immune basis for a subset of IUFD. While the precise systemic and uterine mucosal immune pathways mechanistically responsible for IUFD remain elusive, current research has focused on a role for regulatory T cells (Tregs) in fetal loss.

Tregs are necessary for successful allogeneic mammalian pregnancy (8–13). Evolutionarily, the advent of the placenta coincides with CNS1, the FoxP3 enhancer required for peripheral Treg induction (12). Progesterone acts upon T cells to increase Tregs during implantation, demonstrating immune-hormonal crosstalk in early pregnancy (13). Treg depletion early in gestation or just prior to mating causes complete fetal loss but Treg depletion in mid-late gestation only causes occasional loss (9, 14). The role of Tregs in promoting fetal health in the second half of gestation is unclear.

Research in immune mechanisms of fetal loss has focused on Tregs with fetal-specific T cell receptors (TCRs) and how they may interact with other fetal-specific T cells to maintain tolerance to fetal antigens during pregnancy (8, 11, 15). Tregs are thought to contribute to CD8+ T cell dysfunction in pregnancy as a key mechanism of promoting maternal-fetal tolerance and hence, preventing fetal loss (16–20). While one mechanism of Treg prevention of fetal loss may be inducing tolerance in anti-fetal T cells, Treg depletion in pregnancy is also associated with uterine artery dysfunction and shallow placentation (6, 12, 21, 22). Another potential mechanism by which Tregs prevent fetal loss would be through modulation of uterine mucosal immune populations.

Mucosal immune cells are critical to the protection and homeostasis of barrier sites and are integral to both mucosal disease and their treatment (23–26). In the non-pregnant uterus, mucosal immune cells are implicated in pelvic inflammatory disease, endometriosis, and endometrial cancer. Emphasizing their role in disease pathogenesis, new immune modulatory therapies target these uterine cells (27–31). Most studies on the pregnant uterus have focused on NK cells, which are the most dominant uterine immune population in early pregnancy (32–34). Uterine NK cells defend against congenital infections, are implicated in early pregnancy loss and implantation failure, and have been modulated to treat infertility – demonstrating their multifactorial roles in pregnancy outcomes (35–39). By the second half of gestation, macrophages are more numerous than NK cells in the decidua, but fewer studies have focused the role of decidual macrophages (40).

Human decidual immune cells have also been explored in the setting of pregnancy loss. Multiple studies have compared tissue from first trimester spontaneous abortions and presumed healthy tissue from elective abortions (34, 41–43). While such research is invaluable, human research on fetal loss is limited by when tissue can be ethically obtained: *after* the loss has occurred and generally early in gestation. We therefore seek to address this knowledge gap utilizing an existing mouse model of fetal loss, allowing us to examine decidual immunity across a gestational time course. We hypothesize that the pathophysiology leading to IUFD involves uterine immune cell interactions and that understanding these local cellular dynamics is critical for developing novel therapeutic strategies.

The CBA mouse strain has a reproductive phenotype that is currently characterized as fetal “resorption,” describing a fetal loss pathology with a dark necrotic fetal tissue mass at an implantation site (44, 45). Many studies have demonstrated that immune interventions, such as maternal injections of cytokines, adjuvants, or blocking antibodies alter the rates of fetal loss in CBA mice (46–51). Prior studies have also performed adoptive transfers of Tregs from a variety of sources to decrease rates of CBA fetal loss (21, 44, 52–55). While these studies demonstrate that a systemic immune intervention can impact pregnancy outcomes, they do not identify the cellular immune mechanisms, at the level of the uterus, responsible for fetal loss.

To understand local immune interactions at the maternal-fetal interface contributing to the pathogenesis of IUFD, we utilize the CBA mouse strain. First, we rigorously characterize the reproductive phenotype, interrogate the parental or fetal origin of CBA fetal loss, and examine tissue-specific immune compositions over multiple gestational time points. Second, we queried not only whether Treg adoptive transfer would rescue the fetal loss phenotype, but also how a systemic transfer alters local uterine mucosal immunity. We comprehensively explore uterine tissue immune populations through single-cell RNA-sequencing (scRNA-seq) of uterine myeloid cells from pregnant CBA mice, comparing Treg-recipients to saline control mice. We demonstrate that systemic Treg transfer alters uterine myeloid populations and identify a Trem2+, C1q+, MHC-II^low^ uterine macrophage population, found in both mouse and human data, that may be critical for pregnancy maintenance and reproductive health.

## Results

### CBA mice have multiple aberrant reproductive phenotypes

**Figure 1:**
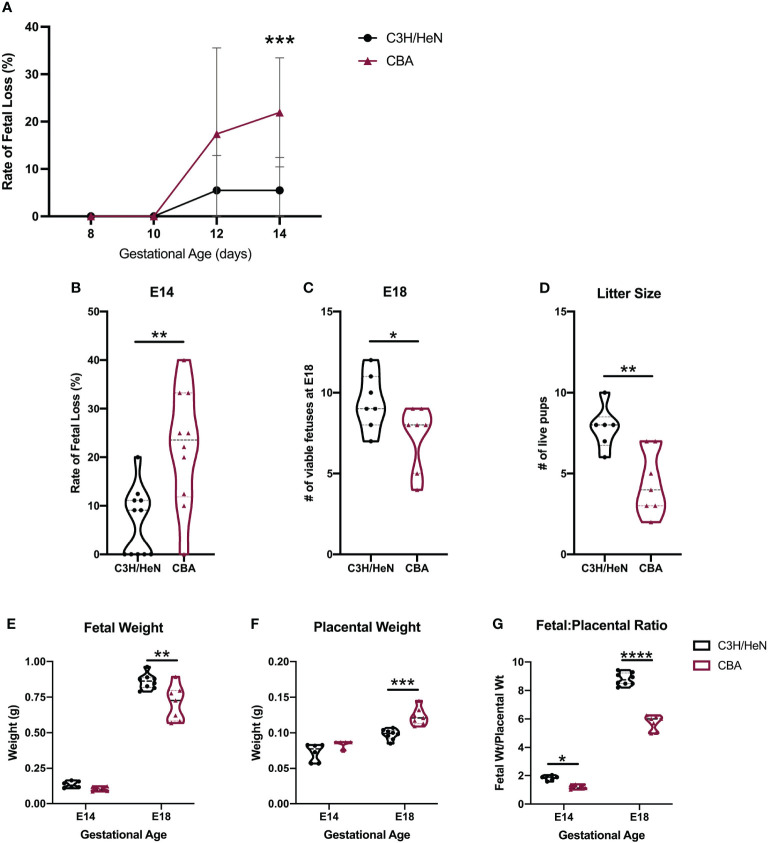
Adverse phenotypes in CBA pregnancy are evident in the second half of gestation. C3H and CBA dams were timed-mated to DBA/2 sires to compare pregnancy phenotypes. **(A)** Pregnant females were sacrificed every two days from E8 – E14 (N = minimum of 4 mice/strain/age) and fetal loss was recorded. In subsequent experiments, **(B)** the rate of fetal loss was reported at E14, **(C)** the number of viable fetuses was counted at E18, and **(D)** the number of live pups was counted on postnatal day 1. **(E)** Fetal and **(F)** placental weights were also recorded on E14 and E18 and the **(G)** fetal-to-placental weight ratio was calculated. For all datasets, normality was assessed by Shapiro-Wilk test. Differences in the rate of fetal loss and fetal and placental weights over multiple gestational ages were assessed by two-way ANOVA, followed by Sidak’s multiple comparison test comparing differences between strains at each gestational age. Differences between two matings at a single gestational age were analyzed by unpaired t-test if normally distributed, or Mann-Whitney test if non-normally distributed. *:p<0.05, **:p<0.01, ***:p<0.001, ****:p<0.0001.

We first characterized CBA reproductive phenotypes by comparing CBA pregnancies to C3H/HeN (C3H) pregnancies. C3H females were used as a control mouse strain because they are close genetic relatives of CBA mice. Both strains were derived from mating a Bagg albino female and DBA male and both have the MHC haplotype H-2^k^ (56). CBA and C3H females were all mated to DBA/2 males. DBA/2 males were selected as a mate because they are the most frequently studied mate of CBA females (45) and are allogeneic from CBA and C3H mice, expressing the MHC haplotype H-2^d^. To determine the timing of fetal loss, CBA and C3H female mice were mated to DBA/2 males and sacrificed every two days from E8 – E14. No evidence of fetal loss was visible at E8 or E10, but was observed from E12 and later gestational ages, indicating a mid-late gestation fetal loss phenotype (**Figure 1A**).

Pregnancies carried by CBA compared to C3H females had higher rates of fetal loss (**Figure 1B**), fewer viable fetuses (**Figure 1C**), and ultimately smaller litter sizes at term (**Figure 1D**). By near term, at E18 – despite a smaller litter size, which usually confers an increase in fetal weight – fetuses from CBA dams weigh less than fetuses from C3H pregnancies (**Figure 1E**). Fetuses from CBA dams also have larger placentae (**Figure 1F**) and a smaller fetal:placental weight ratio (**Figure 1G**) than fetuses from C3H dams, suggesting placental insufficiency. Monochorionic diamniotic twins were also observed in CBA pregnancies but are rarely present in C3H pregnancies (**Figure S1A**). At E14, these twins may both appear normal (**Figure S1B**) or one twin may have demised (**Figure S1C**). These data demonstrate aberrant pregnancy outcomes in CBA mice beyond simply fetal loss, with varying placental phenotypes.

### CBA fetal loss is maternal in origin

**Figure 2:**
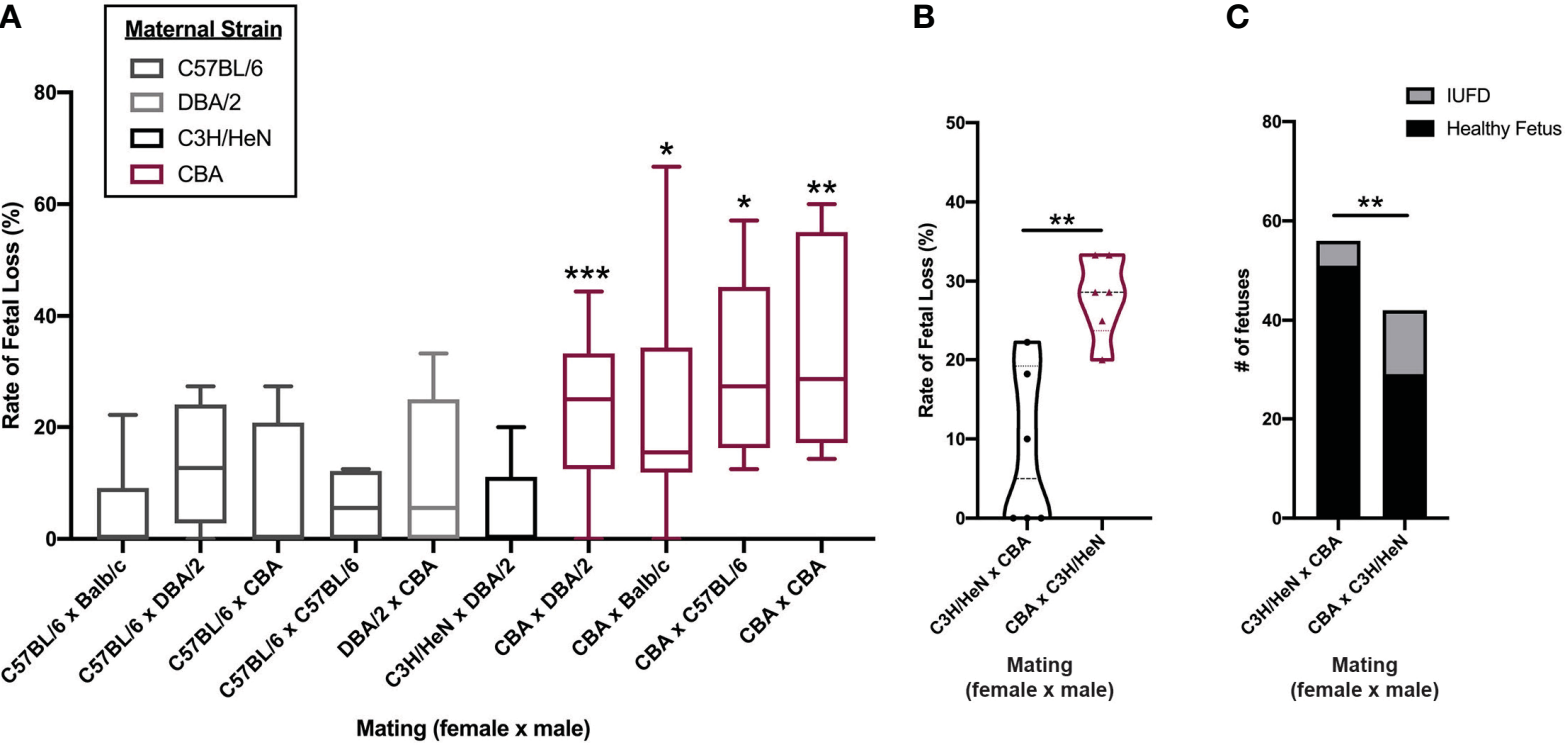
Fetal loss in CBA pregnancy is dependent on maternal strain. Timed-matings were conducted between 5 strains of mice in different mating pairs: C57BL/6, Balb/c, DBA/2, CBA, and C3H/HeN. **(A)** Pregnant dams were sacrificed on E14-15 and healthy versus demised fetuses were counted and reported as a rate of fetal loss. N = minimum of 4 pregnancies/pair. CBA dams were mated with C3H/HeN sires and C3H/HeN dams were mated with CBA sires, side by side. Dams were sacrificed at E14 and healthy versus demised fetuses were counted and **(B)** reported as a rate of fetal loss and **(C)** as a total count of fetuses. Rates of fetal loss among more than two matings were compared by Kruskal-Wallis test followed by a post-hoc Dunn’s multiple comparison test comparing fetal loss in C57BL/6 female x Balb/c male pregnancies to all others. Rates of fetal loss between two matings were compared by Mann-Whitney test. Fetal counts were compared by Fisher’s exact test. *:p<0.05, **:p<0.01, ***:p<0.001.

To determine the mechanism of CBA fetal loss, we first establish whether pregnancy loss is of maternal, paternal, and/or fetal origin. We characterized the pregnancy phenotype by mating both male and female CBA mice to other strains of mice (N = 12 different mating pairs) and comparing the rates of fetal loss to core reference strains, such as C57BL/6 and Balb/c (**Figure 2A**). When CBA male mice sired pregnancies, the rates of fetal loss were the same as seen in more common strains (median rate: 0%) (**Figure 2A**). Conversely, when pregnancies were carried by CBA female mice, the rates of fetal loss were elevated (median rate: 25%) (**Figure 2A**) regardless of paternal strain. As fetal genotype could influence pregnancy outcome, we controlled for this by mating CBA females to C3H males and compared rates of fetal loss to C3H females mated to CBA males. In these pregnancies, the fetal genotypes are the same (CBA x C3H), but the parentage is reversed. In CBA x C3H pregnancies, carried by CBA females, fetal loss was greater than in C3H x CBA pregnancies, carried by C3H females, whether measured as a rate per pregnancy (**Figure 2B**) or as a total count of healthy versus demised fetuses (**Figure 2C**). These data demonstrate that the reproductive phenotype of fetal loss is dependent on the maternal genetic/immune status.

### Pregnant CBA mice have both systemic and local immunologic differences from C3H mice

**Figure 3:**
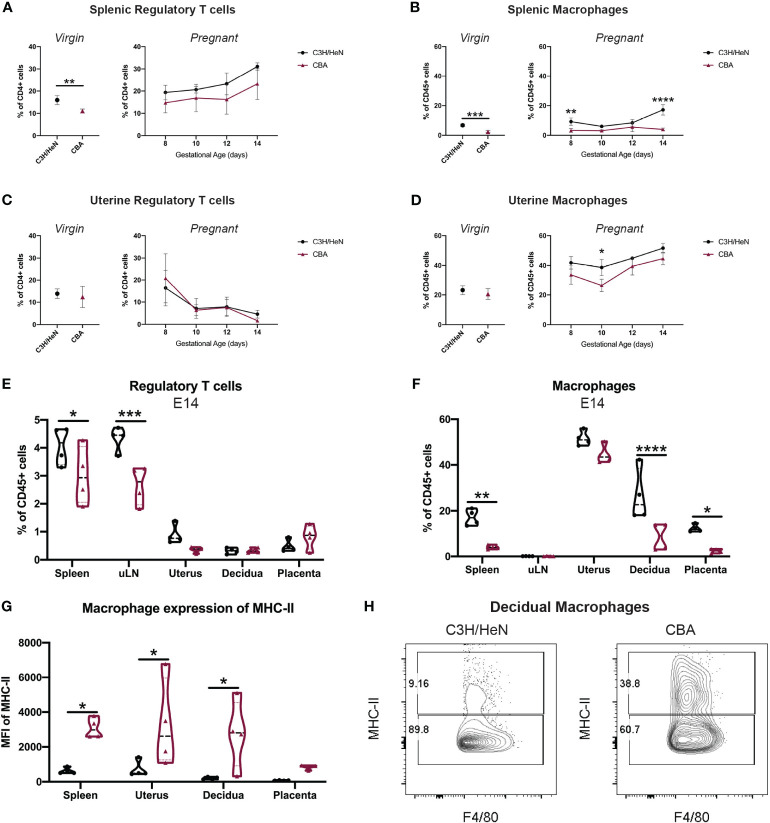
Gestational time course reveals that CBA mice have fewer systemic Tregs and fewer splenic and uterine macrophages. Timed-matings were conducted with CBA and C3H/HeN dams mated to DBA/2 sires. Mice were sacrificed every two days from E8 – E14 and the immune composition of the maternal spleen, uterine-draining lymph nodes (uLN), uterus, decidua, and placentae were analyzed by flow cytometry. Spleen and uterus from virgin CBA and C3H/HeN females were also analyzed. Immune cells in virgin mice (left) and over E8-E14 of gestation (right) are shown for **(A)** splenic Tregs, **(B)** splenic macrophages, **(C)** uterine Tregs, and **(D)** uterine macrophages. At E14, **(E)** Treg and **(F)** macrophage levels, as well as **(G)** MHC-II expression on macrophages are reported in multiple tissues. **(H)** Representative flow plots of decidual macrophages at E14 of C3H/HeN (left) and CBA (right) pregnancy gated on non-debris, singlets, live, CD45+, CD19-, Ly6G-, CD11b+, F4/80+ cells. Immune cell differences in virgin mice were analyzed by unpaired t-test. Gestational time course and comparisons between different organs were analyzed by two-way ANOVA followed by Sidak’s multiple comparison test comparing differences between strain. *p<0.05, **p<0.01, ***p<0.001, ****p<0.0001.

We comprehensively surveyed CBA and C3H immune cell composition in both local and systemic compartments to elucidate CBA immune abnormalities in an unbiased manner. Immune cell composition was first compared in the spleen and uterus of virgin female CBA and C3H mice by flow cytometry using a consistent gating scheme (**Figure S2**). CBA mice have fewer splenic Tregs and macrophages than C3H mice (**Figures 3A, B**). No differences in immune cell composition were detected in the uteri of virgin CBA and C3H female mice (**Figures 3C, D**; **S3**).

To determine if there were local immune differences during pregnancy, CBA and C3H female mice were mated to DBA/2 males and sacrificed every two days from E8 – E14. While some local immune populations followed similar trends over gestation between the strains (**Figures 3C, D**; **S4**), by E14 significant differences were noted in the Treg and macrophage compartments. Spleen and uterine-draining lymph nodes (uLN) of E14 pregnant CBA mice had fewer Tregs than C3H mice (**Figure 3E**). Pregnant CBA mice also had fewer macrophages in the spleen, decidua and placenta, and the CBA macrophages expressed higher levels of MHC-II than C3H macrophages (**Figures 3F, G**). The elevated MHC-II expression on CBA macrophages was notably significant in local uterine and decidual macrophages (**Figures 3G, H**). While no quantitative differences in uterine immune cells were noted between CBA and C3H virgin mice (**Figure S3**), the immune composition of the uterine and decidual environments during pregnancy differ between CBA and C3H mice (**Figures 3B, D–H**), supporting a role for uterine immune cells in the observed reproductive loss in CBA dams.

### Regulatory T cell adoptive transfer prevents fetal loss and decreases MHC-II expression on decidual macrophages

**Figure 4:**
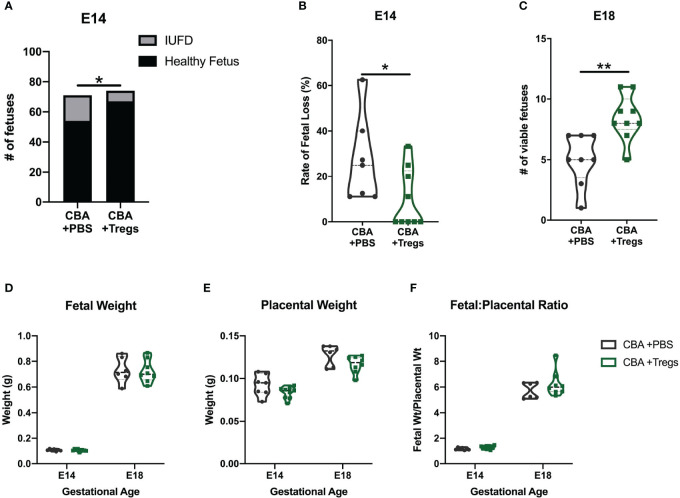
Regulatory T cell adoptive transfer prevents fetal loss. Timed-matings were conducted between CBA dams and DBA/2 sires. At E2-E3, pregnant CBA dams received retroorbital injections of either 2x105 Tregs isolated from the spleen of pregnant C3H/HeN dams or PBS. Mice were sacrificed at E14 and **(A)** healthy versus demised fetuses were counted and **(B)** reported as a rate of fetal loss. In subsequent experiments mice were sacrificed at E18 and **(C)** viable fetuses were counted. The weights of **(D)** healthy fetuses and **(E)** their placentae were measured at E14 and E18 and reported as a **(F)** ratio of fetal weight to placental weight. Count data **(A)** was assessed by fisher’s exact test, fetal loss **(B)** was assessed by Mann-Whitney test, and viability **(C)** was assessed by unpaired t test. Weights **(D–F)** were assessed by two-way ANOVA. *p<0.05, **p<0.01.

Given the systemic deficit of Tregs in both virgin and pregnant CBA female mice and the known importance of Tregs in pregnancy (8–12), we performed a Treg adoptive transfer. Tregs were isolated from spleens of E14 pregnant C3H mice by fluorescence-activated cell sorting (FACS) and 2x10^5^ Tregs in 100μl of PBS were injected into E2 CBA dams. Control CBA mice received 100μl PBS alone. The quantity and timing of Treg transfer is based prior studies of Tregs in CBA mice (44, 53, 54). Maternal Treg transfer increased the numbers of viable fetuses and decreased the rate of fetal loss at E14 and E18 (**Figures 4A–C**). Treg transfer did not increase fetal weight, decrease placental weight, or improve the fetal:placental weight ratio (**Figures 4D–F**). These findings suggest that systemic (maternal) Treg administration modulates a key pathway driving the fetal loss phenotype but appear insufficient to completely reverse the totality of the phenotype.

**Figure 5:**
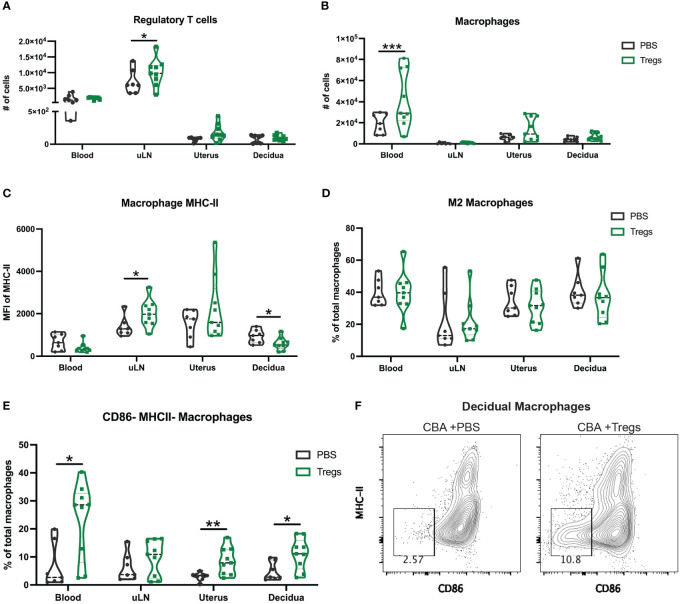
Regulatory T cell adoptive transfer alters the phenotype of uterine macrophages. At E14 following Treg transfer, maternal blood, uterine draining lymph nodes (uLN), uterus, and decidua were collected and analyzed for immune cell composition by flow cytometry including for **(A)** regulatory T cells, **(B)** total macrophages **(C)** macrophage expression of MHC-II, **(D)** M2 macrophages, and **(E)** CD86- MHC-II- macrophages. **(F)** Representative flow plots of CBA decidual macrophages at E14 after Treg transfer (right) and control PBS (left) gated on non-debris, singlets, live, CD45+, CD19-, CD4-, Ly6G-, CD11b+, F4/80+ cells. Two-way ANOVA followed by Sidak’s multiple comparison test was used to compare mice treated with PBS versus Tregs in different organs. *:p<0.05, **:p<0.01, ***:p<0.001. N= min. 6 mice/group.

To assess how systemic administration of immune cells might alter immune composition in the reproductive tissues, we analyzed both systemic and local tissues to determine if the systemic injection of Tregs was able to alter local immune populations in the uterus, decidua, and placenta. We hypothesized that Treg transfer may specifically alter uterine macrophages, given that the primary difference we found between CBA and C3H uterine immune cells was in macrophage expression of MHC-II (**Figure 3G**), and Tregs are known to decrease MHC-II on macrophage cell membranes (57). Pregnant CBA mice, having received Tregs or saline (controls) on E2, were sacrificed on E14, 12 days after the initial injection. Tregs were increased in the uLNs of Treg-recipients over controls (**Figure 5A**). Macrophages were increased in Treg-recipients over controls in maternal blood, but not in the uterus or decidua (**Figure 5B**). Given the lack of quantitative changes in broad immune cell types local to the maternal-fetal interface, we then evaluated macrophage subsets and expression of markers that impact macrophage function. Decidual macrophages from Treg-recipients had reduced expression of MHC-II, similar to decidual macrophages from healthy C3H mice (**Figures 5C**, **3G**). We hypothesized that Treg transfer would induce an ‘M2’-like macrophage phenotype, as has been reported in mice with healthy pregnancy (58), so we analyzed CD206+ MHC-II^low^ macrophages, but did not identify a difference (**Figure 5D**). We then investigated macrophage activation, specifically looking at CD80 and CD86 expression. Treg-recipient mice had more CD86- MHC-II- macrophages in their blood, uterus, and decidua than controls (**Figure 5E**). Decidual macrophage expression of CD86 and MHC-II is visualized in representative flow plots (**Figure 5F**). These results support our hypothesis that a systemic Treg transfer can alter local uterine and decidual macrophages – changing the macrophage phenotype to be more like those from healthy C3H mice.

### Single-cell RNA-sequencing of uterine CD11b+ cells identifies an increase in an uterine macrophage subset following Treg transfer

**Figure 6:**
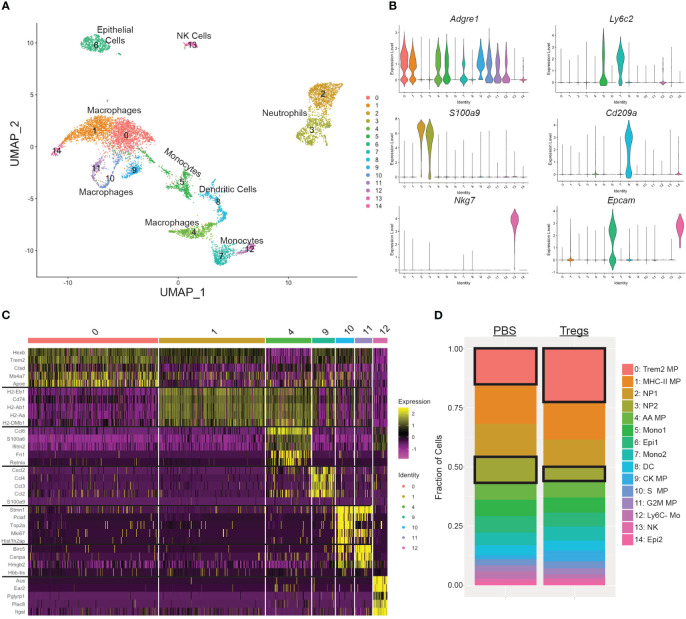
Single cell RNA sequencing of uterine CD11b+ cells identified an increase in Trem2+ macrophages and a decrease in CCL3+ neutrophils following Treg transfer. **(A)** Seurat UMAP clustering of uterine CD11b+ cells combined from all 6 mice identified 15 cell subsets. **(B)** Cell type of each cluster was identified by expression of cell type-defining genes: Adgre1 for macrophages and monocytes, Ly6c2 for monocytes, S100a9 for neutrophils, Cd209a for dendritic cells, Nkg7 for NK cells, and Epcam for epithelial cells. **(C)** Clusters that were Adgre1+ but Ly6c2-(0, 1, 4, 9, 10, 11, 12) were further identified by their individual gene signatures. The top 5 differentially expressed genes for each of those clusters are shown in a heatmap. **(D)** The cellular make up was differentiated between mice that received Tregs versus control mice that received PBS and analyzed by Fisher’s Exact Test. Cluster 0 and cluster 3 had significantly different proportions in the Treg transfer recipient mice. MP, macrophage; NP, neutrophil; AA, alternatively activated; Mono, monocyte; DC, dendritic cell; CK, cytokine. Mo, monocyte; NK, natural killer cell; Epi, decidual epithelial cell.

Our initial Treg transfer demonstrated that a single systemic injection early in pregnancy (E2) could change uterine myeloid populations and sustain those changes for at least 12 days. To investigate specific changes in the uterine myeloid cells induced by a maternal Treg injection, we performed single-cell RNA-sequencing (scRNA-seq) on CD11b+ cells isolated from the uteri of 3 Treg-recipient mice and 3 PBS-injected control mice. The combined sequenced cells were visualized in 2D space by UMAP and 15 clusters were identified (**Figure 6A**). Cluster identities were determined by expression of cell type defining genes (**Figure 6B**). We focused our analysis on macrophages due to our findings by flow cytometry. We identified seven macrophage clusters, expressing *Adgre1* but not *Ly6c2*, indicating they are in the macrophage/monocyte lineage but not classical monocytes: clusters 0, 1, 4, 9, 10, 11, and 12 (**Figure 6B**). Those macrophage clusters were further analyzed for differentially expressed genes (**Figure 6C**). Within the same cell type cluster, there was little differential gene expression between the cells from Treg-recipient or PBS-injected control mice (**Figure S5**; **Table S1**). However, Treg transfer influenced the proportions of each cell type present in the uterus, specifically increasing cluster 0 Trem2+ macrophages (p=2.2e-12) and decreasing cluster 3 neutrophils (p=3.6e-12) (**Figure 6D**).

**Figure 7:**
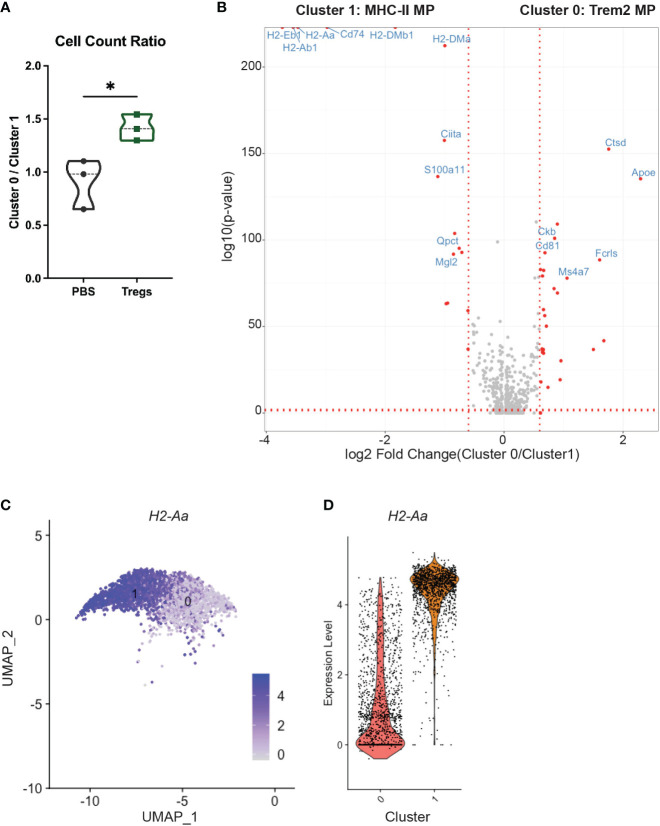
An MHC-II low macrophage cluster is enriched in the uteri of Treg-recipient mice. **(A)** Treg recipient mice have an increased ratio of cluster 0 to cluster 1 cells compared to control mice. **(B)** Volcano plot compares differential gene expression of cluster 0 and cluster 1. **(C)** Feature plot show expression of H2-Aa in clusters 1 and 2. **(D)** Violin Plots enumerate H2-Aa expression in clusters 1 and 2. Differences in cell counts were analyzed by unpaired t-test. *:p<0.05. N = 3 mice/treatment group.

Furthermore, Treg transfer shifted the ratio of the two largest groups of uterine macrophages: cluster 0 and cluster 1. Treg recipients had a 55.2% higher ratio of clusters 0:1 than PBS-injected control mice (**Figure 7A**). Cluster 1 has increased differential expression of MHC-II and related genes (i.e. *H2-Aa*, *H2-Ab1*, *H2-Eb1*, *Cd74*, *H2-DMa*, *H2-DMb1*) when compared to cluster 0, which are visible in the top left corner of the volcano plot (**Figure 7B**). This contrast in MHC-II expression between cluster 0 and cluster 1 can be visualized by examining expression of the class II gene *H2-Aa* between cluster 0 and cluster 1 (**Figures 7C, D**).

### Analyses of uterine macrophages enriched by Treg transfer implicate C1q complex and Trem2 as functionally important genes and proteins

**Figure 8:**
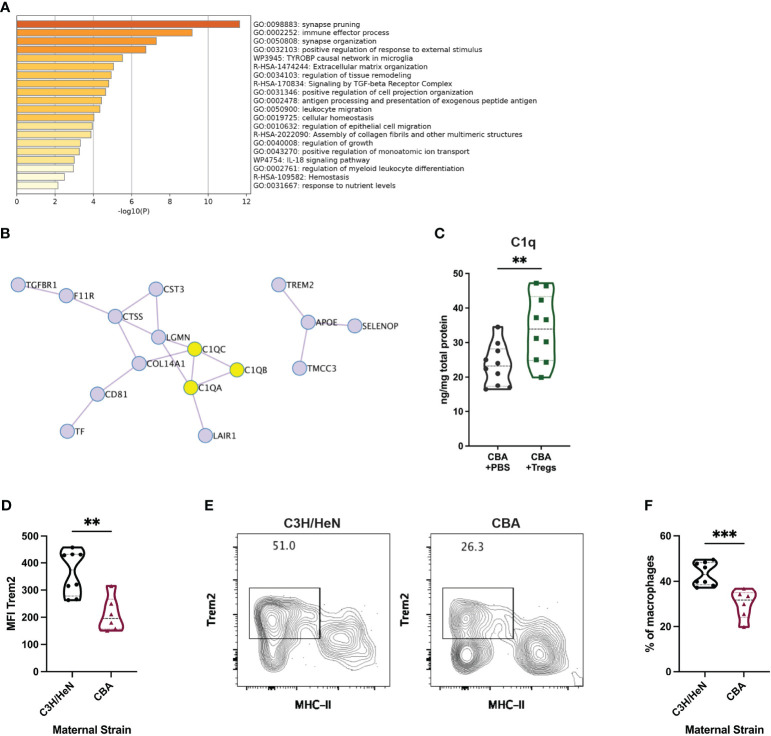
Transcriptional and protein-based analyses implicate C1q and Trem2 as functionally important in uterine macrophages associated with healthy pregnancy. Metascape pathway analysis was completed for the top 50 differentially expressed genes in cluster 0. **(A)** The top 20 enriched pathways are listed. **(B)** Included in those top 50 genes are 16 genes encoding interacting proteins. C1q complex proteins are highlighted in yellow. **(C)** C1q was measured by ELISA from uterine implantation sites of E14 pregnant CBA mice after Treg adoptive transfer or control PBS transfer. C1q protein was normalized to total protein in each sample. Flow cytometry was used to identify Trem2+ MHC-IIlow/neg uterine macrophages from E14 pregnant C3H/HeN (healthy) and CBA (IUFD-prone) dams mated with DBA/2 sires. **(D)** Total macrophage Trem2 expression was measured. **(E)** Representative flow plots were gated on non-debris, singlet, live, CD45+, CD3-, Ly6G-, CD11b+, F4/80+ cells. **(F)** The frequency of Trem2+ MHC-IIlow/neg macrophages was measured. Differences were assessed by unpaired t-test. **:p<0.01; ***:p<0.001.

To investigate potential functions of the cluster 0 uterine macrophages, we conducted pathway analysis on the top 50 cluster defining genes (**Table S2**) (59). Cluster 0 macrophages are enriched for gene pathways including immune effector processes, regulation of response to external stimuli, and synapse organization (**Figure 8A**). The top 50 cluster 0-defining genes encode proteins with many potential interactions, including the C1q complex, transmembrane signaling proteins, and regulation of MHC-II expression (**Figure 8B**). To further analyze potential functions of cluster 0 macrophages, we compared their gene set to known mouse myeloid gene signatures. Gene lists were compiled from scRNA-seq experiments on mouse myeloid cells into a single gene matrix of 123 gene lists (60–72). 47 of the gene lists were upregulated in a ranked list of all genes expressed by cluster 0 uterine macrophages. The normalized enrichment score (NES) of each of these 47 gene lists was plotted against the nominal p-value of the enrichment (**Figure S6A**). Of those upregulated gene lists, 10 had significant nominal p-values of <0.01 (**Figure S6B**). The gene set with the highest NES and lowest false discovery rate (FDR) q-value was one for tumor-associated macrophage 2 (TAM2) from a mouse model of glioblastoma multiforme (GBM) (**Figure S6B**, purple circle). The similarity between the TAM2 gene set and our cluster 0 macrophages can be visualized by an enrichment plot (NES = 1.62, FDR q-value = 0.001) (**Figure S6C**). Leading edge analysis was performed on the 10 gene lists enriched in cluster 0 macrophages with a p-value <0.01 to search for a conserved core gene signature among these macrophage subtypes. Of these leading-edge genes, seven were present in 9 out of 10 gene lists: *C1qa, C1qb, C1qc, Grn, Itgb5, Lgmn*, and *Trem2*. This analysis suggests an important conserved role for those 7 genes in this macrophage subset.

Given the repeated identification of C1q complex and Trem2 in our scRNA-seq analyses, we asked whether these proteins were enriched in healthy pregnancies, in addition to the RNA transcripts. As C1q is a soluble protein, we investigated whether the Treg-induced transcriptional elevation in C1q genes corresponded to an increase in soluble protein. Uterine implantation sites of pregnant CBA mice that received a Treg adoptive transfer had 44.7% more C1q protein than control mice as measured by ELISA (**Figure 8C**). Uterine macrophages from pregnant C3H mice, without any known reproductive pathology, expressed 1.7-fold more Trem2 than those in CBA mice (**Figure 8D**). A clear population of Trem2+ MHC-II^low/neg^ macrophages was identified by flow cytometry (**Figure 8E**) and healthy C3H mice had 44.8% more uterine Trem2+ MHC-II^low^ macrophages compared to the IUFD-prone CBA mice (**Figure 8F**).

### The gene signature of C1q+ Trem2+ mouse uterine macrophages is present in human decidual macrophages

**Figure 9:**
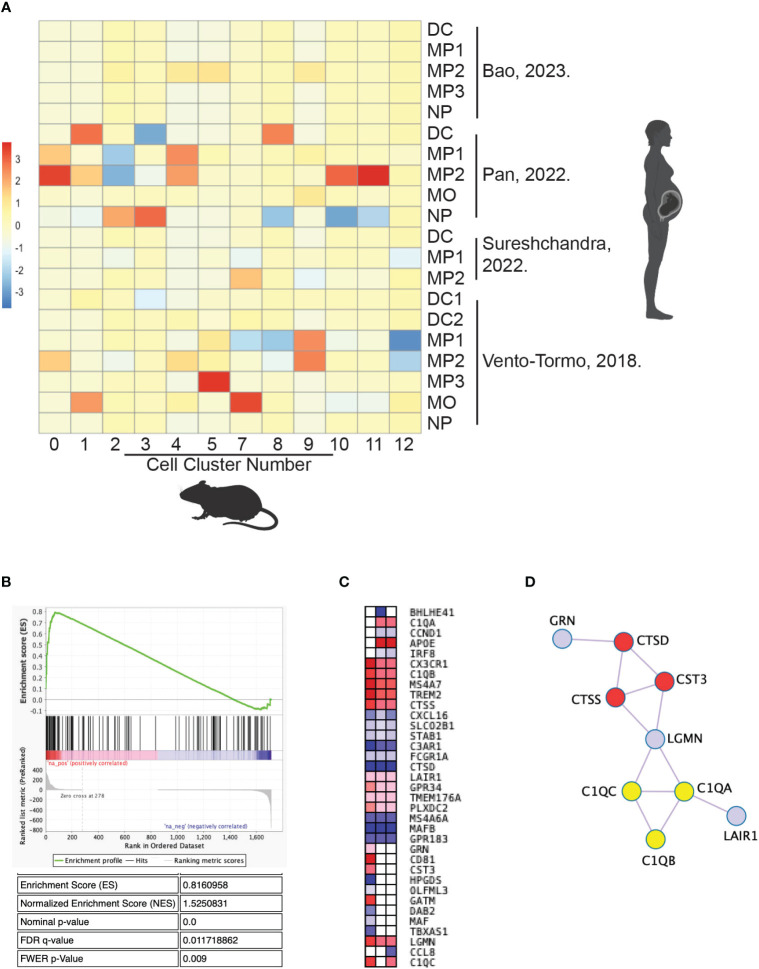
Cluster 0 mouse macrophages share a conserved gene signature with human decidual macrophages. Published data of scRNA-seq from human decidual myeloid cells were compared with scRNA-seq data in this study of mouse uterine myeloid cells by gene set enrichment analysis. **(A)** The enrichment score of each mouse cluster gene set for each human cell gene list is graphed in a heat map. **(B)** The gene set enrichment plot for human decidual macrophage 2 gene list from Pan, et al. in mouse uterine cluster 0 macrophages. **(C)** Leading edge genes of the three human gene lists with p-values <0.05 for enrichment in cluster 0 macrophages. **(D)** Protein interactions identified within the leading edge gene set. C1q genes are marked in yellow and peptidase-related genes are marked in red. DC, dendritic cell; MO, monocyte; MP, macrophage; NP, neutrophil.

Given the biologic differences between mice and humans, we assessed whether the identified myeloid subtypes in the uteri of CBA pregnancies were present in normal human decidua. To our knowledge, three groups have published scRNA-seq data on immune cells from early pregnancy maternal-fetal interface tissues, including decidua from elective terminations and spontaneous abortions (34, 41, 73). A fourth group has published scRNA-seq data from postpartum term decidua (74). Using gene set enrichment analysis, the macrophage and monocyte populations from our murine gene set were compared to those in the published human gene sets (**Figure 9A**). The myeloid populations from the mouse scRNA-seq gene sets matched nearly 1:1 with a myeloid population in a human decidual scRNA-seq gene set (**Figure 9A**). Cluster 0 mouse macrophages were most enriched in a human decidual macrophage from Pan, et al. (NES=1.53, FWER p-value=0.009) (**Figures 9A, B**) (34). The core enriched genes between the two macrophage populations include *C1QA, C1QB, C1QC*, and *TREM2* in the top 7 genes (**Table S3**).

Of note, there was minimal overlap between the mouse clusters and cell types from Bao, et al. or Sureshchandra, et al. (**Figure 9A**) (41, 74). Bao et al. used cell isolation methods to enrich for stromal cells in their scRNA-Seq experiment, while the other three groups intentionally enriched for immune cells with methods akin to ours. Sureshchandra et al. is the only group to analyze postpartum decidual tissue, demonstrating the immunological difference in tissue collected after the relatively inflammatory process that is labor.

Nonetheless, Cluster 0 macrophages shared statistically significant (nominal p-value <0.05) transcriptional overlap with three human decidual macrophage populations: MP1 and MP2 from Pan et al. and MP2 from Vento-Tormo, et al. (**Figure 9A**) (34, 73). Leading edge analysis of those three human gene sets was performed to identify a core gene signature for this cell type (**Figure 9C**). *C1QA, C1QB, C1QC*, and *TREM2* were 4 of 13 genes positively enriched in at least two gene lists, redemonstrating the likely functional importance of those genes in both human and mouse uterine macrophages (**Figure 9C**). Many of the leading-edge genes encode for interacting proteins, including those for the C1q complex and peptidase-related genes (**Figure 9D**). Thus, the enriched genes between cluster 0 mouse uterine macrophages and human decidual macrophages resemble a conserved gene signature, further supporting that this group of genes are critical to the function of these uterine macrophages and suggesting interspecies conservation of this cell type in pregnancy.

## Discussion

We have identified a maternal-derived mechanism of fetal loss associated with both a deficit in maternal systemic Tregs and aberrant uterine myeloid cells. These data confirm our hypothesis that systemic Tregs alter local immune cells at the maternal-fetal interface as one mechanism to prevent fetal loss. Furthermore, for potential translational impact, these data demonstrate that a systemic intervention to the mother can alter tissue-specific uterine immune populations. Using scRNA-seq, we discovered a novel Trem2+ C1q+ MHC-II^low^ uterine macrophage population, present in mouse and human decidua, that may be necessary for optimal reproductive health. Collectively, these data demonstrate a novel mechanism by which Tregs influence pregnancy outcomes through modulation of the uterine immune composition, particularly macrophages, which impacts pregnancy viability and fetal health.

The CBA strain has been utilized to study recurrent pregnancy loss (RPL) (51, 75–79). However, there have been concerns that the reproductive phenotype of CBA pregnancy loss does not sufficiently recapitulate human RPL (45). Our rigorous approach enabled us to characterize the reproductive phenotype of CBA pregnancy more comprehensively. In performing a detailed analysis over gestation, we demonstrate that fetal loss in the CBA model is post-implantation and is specific to the second half of pregnancy (**Figure 1A**). As such, the reproductive phenotype in CBA pregnancy is most consistent with the clinical scenario of IUFD than early spontaneous abortion. This conclusion is consistent with findings of uterine and placental vasculature abnormalities in mid-gestation CBA mice, showing that the uterine arteries have small lumen-to-wall ratios and placentae have evidence of thrombosis and fibrin deposition (76, 78). Placentae from human IUFDs often have similar findings and placental pathology is considered the most useful test to help determine causes of IUFD (80). Therefore, our data on the timing of fetal loss (**Figure 1A**) and placental abnormalities (**Figures 1E–G**; **S1**) in the CBA strain is consistent with human IUFD.

Treg adoptive transfers have been previously performed on CBA mice with a focus on the impact of Tregs on early implantation, Treg antigen-specificity, or *in vitro* Treg induction strategies (21, 44, 53, 54). Although we use Tregs from C3H mice rather than CBA mice, our findings reproduce the existing data that Tregs reduce the rate of fetal loss in CBA mice. These prior studies have primarily focused on the function of Tregs themselves: cytokine production, response to paternal alloantigen, suppressive activity, or epigenetic modifications (44, 53, 54); or Treg impact on implantation (14, 21). In contrast, we interrogated how an increase in maternal Treg number would affect reproductive tissues by examining tissue resident immune cells at the time of fetal loss. Decidual macrophage polarization has been shown to impact pregnancy outcomes, with more immunosuppressive or “M2-like” macrophages associated with healthy pregnancy (58, 81–83). Tregs are known to induce a pro-phagocytic, anti-inflammatory phenotype in macrophages (57, 84). Our data provides a mechanistic link between systemic Tregs and uterine macrophage polarization.

The cluster 0, MHC-II^low^ uterine macrophages increased by Treg transfer highly transcribe the TGFβ receptor gene (*Tgfbr1*) and “signaling by TGF-beta receptor complex” is one of the key pathways identified in their gene signature (**Figure 8A**). These findings suggest that Tregs may communicate to MHC-II^low^ uterine macrophages via TGFβ. Communication between Tregs and macrophages via cytokines, rather than MHC-II : TCR interactions, induces a macrophage phenotype with reduced MHC-II and increased scavenger receptor expression (57, 84, 85). Low expression of MHC-II implies that cluster 0 uterine macrophages are not presenting fetal antigen to the Tregs. Moreover, the mechanism of CBA fetal loss is likely to be independent of fetal antigen, given that CBA syngeneic pregnancies still have elevated rates of fetal loss (**Figure 2A**). These data support antigen-independent manner of Treg manipulation of macrophage phenotype, leading to improved pregnancy outcomes.

An antigen independent mechanism of fetal loss does not suggest that MHC-II plays no role in CBA fetal loss. To the contrary – our data demonstrate that a low ratio of cluster 0 (MHC-II^low^) to cluster 1 (MHC-II^high^) macrophages in the uterus is associated with fetal loss (**Figure 2A**). The top five cluster-defining genes for cluster 1 macrophages are all MHC-II related genes (**Table S2**). This is consistent with data showing that macrophages at the maternal-fetal interface in healthy pregnancy often have more MHC-II surface expression later in pregnancy (40).

One potential role of MHC-II^low^ uterine macrophages is to clear cellular debris, which accumulates during trophoblast invasion and placentation (86, 87). Tregs induce efferocytosis in macrophages (57, 84) and increased efferocytosis of apoptotic trophoblasts by MHC-II^low^ macrophages may decrease recognition of trophoblasts by MHC-II^high^ macrophages. Twelve of the top 20 cluster-defining genes for cluster 0 macrophages are associated with phagocytosis and breaking down molecules in the endosomal and lysosomal systems, consistent with efferocytosis and other phagocytic processes as key functions (**Table S2**). Within those twelve, Trem2 and C1q genes were enriched in every analysis of the cluster 0 gene signature (**Figures 6C**, **8B**, **9C, D**; **S6B, C**). Both Trem2 and C1q have roles in phagocytosis and removal of aberrant cellular material, which may be necessary for maternal-fetal tolerance. Trem2 in microglia prunes synapses and clears dying neurons (88, 89). C1q has been studied more specifically in pregnancy and C1q-deficient mice have pathologic pregnancies with fetal loss, insufficient trophoblast invasion, and kidney damage (90–93).

In human pregnancy, endovascular trophoblasts express C1q-receptor and bind to C1q expressed on the surface of decidual endothelial cells (91, 94). Human decidual dendritic cells and macrophages also express C1q (87). Patients with preeclampsia have reduced serum levels of C1q (95), which supports our finding of reduced C1q in entire implantation sites of IUFD-prone mice as indicative of poor pregnancy outcomes (**Figure 8C**). The interactions between endovascular trophoblasts expressing C1q-receptor and decidual cells expressing C1q are thought to be critical for spiral artery remodeling, which when defective can lead to IUFD and preeclampsia (92, 93).

While C1q may be critical for decidual vascular remodeling, Trem2 may support tissue invasion. Trem2+ uterine macrophages contribute to the migration of endometriotic epithelial cells in endometriosis (96). Furthermore, the cluster 0 MHC-II^low^ uterine macrophages identified in this study transcriptionally resemble tumor-associated macrophages (TAMs) (**Figures S6B, C**). Trem2+ MHC-II^low^ TAMs are immunosuppressive and promote tumor survival and growth (70), and likewise Trem2+ MHC-II^low^ uterine macrophages may also be immunosuppressive and promote fetal survival and growth. The process of placentation, of trophoblast invasion into the uterine vasculature, has been fittingly described as akin to tumor invasion into tissue vasculature (97), and therefore MHC-II^low^ uterine macrophages may be necessary for successful placentation.

Both Trem2 and C1q are drug targets in preclinical trials. Gene therapies promoting overexpression of Trem2 are being investigated for treatment of Alzheimer’s Disease and other neuroinflammatory disease, while Trem2 neutralizing antibodies are being studied as a potential adjuvant to immunotherapy for solid tumors (98–101). C1q inhibitors are in clinical trials for complement-mediated autoimmune disease and recombinant C1q is in preclinical investigation for decreasing inflammatory responses to influenza and reprogramming macrophages ex-vivo in autoimmunity (102–105). Given that both proteins are already targets of drug development, they may be feasibly modulated to impact obstetric complications. Based on this study, therapeutic strategies that increase Trem2 and C1q levels are likely to be effective for prevention of IUFD.

In addition to therapies promoting Trem2 and C1q, many clinical trials are investigating methods to increase systemic Tregs to treat autoimmune disorders and prevent rejection of transplanted organs (106–109). These trials have studied both cellular therapy of autologous *in vitro* expanded Tregs and immunotherapies such as teplizumab and aldesleukin to expand patient Tregs *in vivo* (106–109). In obstetrics, direct intrauterine infusion of Tregs has been studied for treatment of RPL (110). Other studies have suggested Treg-directed therapies could prevent the inflammation causing preeclampsia and preterm labor, but these have not yet been clinically tested (111, 112). Our model suggests that expansion of systemic maternal Treg protects from fetal loss, indicating Treg-targeted therapies may be beneficial for patients at risk for IUFD.

Because of biological differences between murine and human pregnancy (e.g. multifetal pregnancy, short gestation), there are some limitations to the use of the CBA mouse strain in investigating reproductive outcomes. However, noting these limitations, mouse models have provided critical insight into mechanisms of reproduction and parturition (11, 19, 113, 114). Additionally, mechanistic studies on reproductive tissues over gestation are not feasible in humans and collection of fetal and maternal tissues from mid-late gestation human pregnancies is not ethical. While tissues may occasionally be available after diagnosis of an IUFD, these tissues cannot be informative as to the tissue or immune dynamics that occurred prior to the loss. Notably, many mouse decidual myeloid cells from our data shared gene signatures with early gestation human decidual myeloid cells identified by Pan, et al. and Vento-Tormo, et al. (**Figure 9A**), suggesting a conserved cell functions across species (34, 73).

An additional limitation to the CBA mouse is that the phenotype is complex and likely mediated by multiple factors, including those that are environmental or microbial. For example, in other studies CBA females mated to Balb/c sires have less fetal demise than with DBA/2 sires (44, 47, 115). However, we found no difference in the resorption rate between CBA females mated to Balb/c (22.8% resorption rate) or DBA/2 (25.1% resorption rate) sires (N=14 matings of each parentage; p=0.719). Additionally, Treg transfer had no effect on placental and fetal size abnormalities (**Figures 4D-F**), indicating that Tregs are not fully reversing the CBA reproductive phenotype.

A limitation of our scRNA-seq experiment is that we only examined one time point during gestation and compared a Treg transfer to saline control without using an additional effector T cell control. While this gestational age was carefully chosen based on our time course experiments and the occurrence of the reproductive phenotype in the CBA strain, other key differences induced by Tregs prior to E15 might have been missed. For this study, we prioritized having three mice per group and comparing the Treg intervention to a non-intervened control. Future research could elucidate more precise timing as to how rapidly Treg injection can alter uterine immune cells and the potential immunological impact of effector T cell injection.

To our knowledge, this is the first study to report findings from scRNA-seq on the myeloid populations of a pregnant mouse uterus, and thus contributes to the growing field of reproductive immunology. These results build upon research that has characterized specific immune cells present at the maternal fetal interface using flow cytometry, immunohistochemistry, and cell type-deficient mice (32, 97), by adding unbiased and thorough transcriptomic information. Single-cell experiments have been conducted on human tissue from the maternal-fetal interface (34, 41, 73, 74, 116), and these studies have provided valuable insight into the normal immune composition of the decidua and placenta. However, human studies are limited to postpartum or aborted tissues, limiting their application to IUFD and other events in mid-late gestation.

While there have been large cohort studies performed to address IUFD, there remains little mechanistic data on human IUFD (117). This study leveraged scRNA-seq technology to identify a key cell type and targetable pathways for therapeutics for preventing IUFD. Future studies can leverage these findings to explore how identification and treatment of these aberrant immune responses in human pregnancy may be a possible new avenue to predict and prevent IUFDs. Our data most suggest that a systemic cell-based therapy can have a lasting functional impact on tissue resident immune cells. This discovery has implications not only for treating reproductive pathologies but also to all disease caused by tissue-specific immune populations.

## Materials and methods

### Animals

Timed-pregnant mice were mated in our mouse colony and vaginal plugs were checked to confirm copulation. Plugged females were removed from male cages. Mice were euthanized by CO_2_ at the indicated gestational age. All animal care and use procedures are approved by the University of Pennsylvania IACUC.

### Tissue collection

At the indicated gestational age, mice were euthanized, and tissues were collected into and washed with 10% charcoal-stripped fetal bovine serum (FBS; Gemini Bio, Sacramento, CA) in Hank’s balanced salt solution (HBSS) on ice. Maternal spleen and uterine-draining para-aortic lymph nodes (uLN) were collected. The uterus was bisected and gestational sacs with fetal membranes and placentae were removed. The decidua was then scraped from uterus with a glass microscope slide into HBSS + 10% FBS on ice. Membranes were removed from the placenta and the placenta and uterus were washed in HBSS + 10% FBS on ice. Tissues were immediately processed into a single cell suspension for flow cytometry.

### Single cell suspension

Preparation of tissues for flow cytometry follows a protocol established with the placenta (118). Placentae and uterus were minced into 2mm pieces, suspended in 5ml of digest solution (HBSS + 10% FBS + 1mg/ml Collagenase IV, Gibco, Gaithersburg, MD), and incubated in a 37°C water bath for 30 minutes. Placentae and uterus (post-digest treatment), maternal spleens, uLN, and decidua were mechanically pressed through a 70μm cell strainer. The strainer was rinsed with 10ml of HBSS and strained cells were passed through a 40μm cell strainer into a 50ml conical tube. Cells were centrifuged at 1500rpm, 4°C for 10 minutes. Red blood cells were eliminated from tissues by suspension in 3ml of ACK lysing buffer (Gibco, Gaithersburg, MD) and incubation at room temperature for 10 minutes. ACK-treated cells were rinsed in 10ml of HBSS and centrifuged at 1500rpm, 4°C for 5 minutes. Single cell suspensions from maternal spleen, uLN, decidua, uterus, and placenta were suspended in 1ml of FACS buffer (PBS + 2% FBS + 20μM EDTA) and transferred into 5ml round-bottom tubes through 35μm filter caps. Cells were stored on ice until ready for staining.

### Flow cytometry staining

Cells were transferred to a 96-well round-bottom plate for staining, washed with PBS, and the plate was centrifuged at 1500rpm, 4°C for 5 minutes. Cells were resuspended in Live/Dead Fixable Aqua (Invitrogen, Waltham, MA) diluted 1:1000 in PBS and incubated for 30 minutes at 4°C in the dark. Cells were washed twice with FACS buffer, centrifuging the plate between washes at 1500rpm, 4°C for 5 minutes. Cells were then resuspended in an extracellular antibody mix and incubated for 30 minutes at 4°C in the dark. Extracellular antibody mixes included: anti-CD3e-APC-Cy7, anti-CD3e-BV785, anti-CD4-BV605, anti-CD4-PE-Cy7, anti-CD11b-PE, anti-CD11b-PerCP-Cy5.5, anti-CD11c-PE-Cy7, anti-CD19-APC, anti-CD19-APC-Cy7, anti-CD45-BV711, anti-CD49b-PacificBlue, anti-CD64-PE, anti-CD86-AF700, anti-CD122-FITC, anti-F4/80-FITC, anti-Ly6C-APC-Cy7, anti-Ly6G-PerCP-Cy5.5, anti-MHC-II-BV785, anti-TCRγ-PerCP-Cy5.5, anti-Tim3-APC, (BioLegend, San Diego, CA); anti-CD8a-APC.efluor780, anti-CD80-SB436, anti-CD206-PE-Cy7, anti-SiglecF-SB600 (eBioscience, San Diego, CA); anti-CD49a-BV605, anti-CD74-BV605 (BD Biosciences, San Jose, CA); anti-Trem2-APC (R&D Systems, Minneapolis, MN). Cells were washed twice with FACS buffer, centrifuging between washes at 1500rpm, 4°C for 5 minutes. Cells were resuspended in fixation/permeabilization reagent (Transcription Factor Staining Buffer Set, eBioscience, San Diego, CA) and incubated for 30 minutes at 4°C in the dark. Cells were washed twice with permeabilization buffer (Transcription Factor Staining Buffer Set, eBioscience, San Diego, CA), centrifuging at 2000rpm, 4°C for 5 minutes between washes. Cells were resuspended in anti-FoxP3-PE (eBioscience, San Diego, CA) diluted 1:300 in permeabilization buffer and incubated for 30 minutes at room temperature in the dark. Cells were washed twice with permeabilization buffer, centrifuging at 2000rpm, 4°C for 5 minutes between washes. Cells were then resuspended in FACS buffer and transferred into 5ml round-bottom tubes through 35μm filter caps and stored at 4°C in dark until analyzed by an LSR-II flow cytometer (BD Biosciences, San Jose, CA).

### Treg adoptive transfer

Spleens were harvested from E13-15 pregnant C3H/HeN mice mated to DBA/2 male mice and processed into a single cell suspension as noted above. CD4+ cells were isolated from splenic cells using a CD4+ T Cell Isolation Kit, mouse (Miltenyi Biotec, Bergisch Gladbach, Germany) following the manufacturer’s instructions. Cells were transferred into 5ml round-bottom tubes through 35μm filter caps, and centrifuged at 300xg, 4°C for 10 minutes. Cells were washed with FACS buffer and centrifuged again. Cells were then stained with extracellular antibody mix: anti-CD8-APC (1:400), anti-CD4-PacificBlue (1:400), and anti-CD25-PE/Dazzle (1:200) (BioLegend, San Diego, CA) in FACS buffer for 30 minutes, 4°C, dark. Cells were washed twice with FACS buffer, centrifuging at 300xg, 4°C for 10 minutes between washes. Cells were resuspended to 2 x 10^7^ cells/ml of FACS buffer. Cells were then sorted by the Penn Flow Cytometry Core staff on a BD FACSAria cell sorter (BD Biosciences, San Jose, CA). Collected Tregs were gated on lymphocytes, single cells, CD8-, CD4+, CD25^high^. These cells were sorted into RPMI media with 50% FBS, centrifuged at 400xg, at 4°C for 10 minutes and washed with PBS. Tregs were resuspended to 2x10^6^ cells/ml in sterile PBS. Timed-pregnant E2 CBA mice mated with DBA/2 males were anesthetized by isoflurane and then received 100μl of the Treg suspension (2x10^5^ Tregs) or 100μl of sterile PBS by retro-orbital injection. CBA mice receiving Tregs or PBS injections were co-housed until sacrifice on E14 or E18. Results are combined from 2-3 experiments at each time point with N=3-5 dams/group/experiment.

### Isolating CD11b+ uterine cells

Uteri were harvested from E15 timed-pregnant CBA mice that had received a Treg adoptive transfer or PBS injection on E2 (N=3/group) and processed into a single cell suspension as noted above. Uterine cells were then resuspended in 1ml of MACS buffer and CD11b+ cells were isolated with CD11b+ microbeads (Miltenyi Biotec, Bergisch Gladbach, Germany) following the manufacturer instructions. Eluted CD11b+ cells were centrifuged at 300xg, at 4°C for 10 minutes. Cells were then resuspended in Live/Dead Fixable Aqua (Invitrogen, Waltham, MA) diluted 1:1000 in PBS and incubated for 30 minutes at 4°C in the dark. Cells were rinsed twice with FACS buffer, centrifuging at 300xg, 4°C for 5 minutes between washes. Cells were then stained with the following extracellular antibody mix, all diluted 1:400 in FACS buffer: anti-CD45-SB702 (eBioscience, San Diego, CA); anti-CD11b-PE, anti-CD3e-APC-Cy7, and anti-CD19-APC.Cy7 (BioLegend, San Diego, CA). Cells were incubated for 30 minutes at 4°C in the dark, washed twice with FACS buffer, centrifuging between washes at 300xg, 4°C for 5 minutes. Cells were resuspended to 1x10^6^ cells/ml in FACS buffer. Cells were then sorted by the Penn Flow Cytometry Core staff on a BD FACSAria cell sorter (BD Biosciences, San Jose, CA). Collected cells were gated on live, singlet, CD45+, CD11b+ cells and sorted into 50% FBS + 50% PBS. 15,000 collected cells/sample were spun down and resuspended in 15μl sterile PBS and brought to the University of Pennsylvania Next Generation Sequencing Core.

### Single-cell RNA-sequencing

Library preparation and sequencing was performed by the University of Pennsylvania Next Generation Sequencing Core. Cell libraries were prepared using 10X Genomics Single Cell Controller and libraries were sequenced on an Illumina HiSeq 2500. Transcriptomic matrices were created with 10X Genomics’ Cell Ranger Single Cell Software Suite 3.1.0. Raw base call files from the HiSeq 2500 sequencer were demultiplexed with the Cell Ranger “mkfastq” pipeline into library-specific FASTQ files. All library-related FASTQ files were subsequently transformed into count matrices with Cell Ranger’s “count” pipeline *vs* genome reference mm10-3.0.0 (Ensembl 93). The retained reads were quantified and used to generate feature-barcode matrices.

### Seurat analysis

Resulting matrices for each sample (N=3/group) were merged into a single object with the merge function in Seurat version 3.1.5 (119). Within this dataset, cells with more than 200 genes and less than 2500 genes were retained, provided the cell did not express more than 10% of genes derived from the mitochondria. The reduced dataset was then normalized and scaled using Seurat default methods. UMAP clustering was performed using Seurat with a clustering resolution of 0.5 and 75 principal components. Gene lists for each cluster were created using “FindConservedMarkers” (**Supplementary Table 2**). Seurat was also used to identify differentially expressed genes in each cluster between PBS and Treg-recipient mice using the “FindMarkers” command (**Supplementary Table 1**).

### Gene set analysis

Gene signatures for each UMAP cluster were analyzed by the Gene Set Enrichment Analysis (GSEA) application from the Broad Institute (120). Gene signatures were compared to gene matrices from the Molecular Signatures Database, including hallmark gene sets, ontology gene sets, and immunologic signature gene sets. Very few of these gene sets were enriched in the clusters of interest, so a gene matrix was created. Gene lists were compiled from papers using scRNA-sequencing on mouse myeloid cells published in the last 5 years, where full gene lists were published in **Supplementary materials** (rather than raw sequencing data). A single gene matrix was created with these 123 gene lists and was compared to the gene signatures from UMAP defined clusters with the Broad GSEA application (version 4.1.0). Metascape pathway analysis was also used to identify cellular pathways for each cluster, specifically by searching the top 50 cluster defining genes (59).

### C1q ELISA

Implantation sites from CBA mice post-adoptive transfer to be used for ELISA were flash frozen in liquid nitrogen at the time of tissue harvest and were stored at -80°C. Protein extracts were made implantation site tissue by submerging 50mg of frozen tissue in 1ml of RIPA buffer with cOmplete mini protease inhibitor cocktail (Roche, Basel, Switzerland) in a 2ml round bottom tube with a 5mm steel bead. Tissue was homogenized on Tissue Lyser II (Qiagen, Venlo, Netherlands) for 10 minutes at 30/second. Homogenate was rested on ice for 40 minutes and then centrifuged at 14000xg for 10 minutes. Total protein in supernatant was quantified by the BCA protein assay kit (Pierce, Rockford, IL) following the manufacturer’s protocol. Mouse C1q ELISA kit (Abcam, Cambridge, UK) was used to measure C1q protein levels, following the manufacturer’s protocol. C1q levels were normalized to total protein.

### Analysis of human dataset

Droplet-based single-cell data from Vento-Tormo, et al. was downloaded from the EMBL-EBI ArrayExpress database (E-MTAB-6701) (73). Feature-barcode matrices and annotation level data was read into the R computing environment. The R package, Seurat, was used for secondary analysis of the external dataset (119). Briefly, cell type annotations were used for grouping/differential expression analysis using dM1, dM2, and dM3 cell types. Differentially expressed gene lists were generated using a Wilcoxon rank sum test. Adjusted p-values were based on a Bonferroni correction using all features in the dataset.

### Statistics

Prism (GraphPad, San Diego, CA) was used for statistical analyses. All datasets were analyzed for normality by Shapiro-Wilk test. Non-normally distributed data with two groups was analyzed by Mann-Whitney test. Normally distributed data with two groups was analyzed by unpaired student’s t-test, with Welch’s correction applied if the two groups differed in variance by F test. Time course data was analyzed by two-way ANOVA, followed by *post-hoc* Sidak’s multiple comparison test comparing CBA to C3H immune cells at each gestational age. Proportion data was analyzed by Fisher’s Exact test.

### Study approval

All animal care and use procedures are approved by the University of Pennsylvania IACUC.

## Data availability statement

The single-cell RNA sequencing data presented in this study are deposited in the Gene Expression Omnibus (GEO), accession number GSE244616. Gene lists from this analysis can be found in the **Supplementary Material**.

## Ethics statement

The animal study was approved by University of Pennsylvania Institutional Animal Care and Use Committee. The study was conducted in accordance with the local legislation and institutional requirements.

## Author contributions

EL: Conceptualization, Data curation, Formal Analysis, Investigation, Methodology, Writing – original draft. ER: Data curation, Formal Analysis, Writing – review & editing. LA: Conceptualization, Investigation, Project administration, Writing – review & editing. MG: Formal Analysis, Writing – review & editing. DT: Supervision, Writing – review & editing. PP: Conceptualization, Funding acquisition, Methodology, Supervision, Writing – review & editing. ME: Conceptualization, Funding acquisition, Methodology, Resources, Supervision, Writing – review & editing.

## References

[1] Dongarwar D Aggarwal A Barning K Salihu HM. Trends in stillbirths and stillbirth phenotypes in the United States: an analysis of 131.5 million births. Int J Maternal Child Heal AIDS (2020) 9:146–8. doi: 10.21106/ijma.344 PMC7031879 32123637

[2] Gregory EC Valenzuela CP Hoyert DL. Fetal mortality: United States, 2020. Natl Vital Stat Rep (2022) 71:1–20. doi: 10.15620/cdc:118420 35947824

[3] Man J Hutchinson JC Heazell AE Ashworth M Levine S Sebire NJ. Stillbirth and intrauterine fetal death: factors affecting determination of cause of death at autopsy. Ultrasound Obst Gyn (2016) 48:566–73. doi: 10.1002/uog.16016 27781317

[4] Stillbirth Working Group. Working to address the tragedy of stillbirth. Eunice Kennedy Shriver Natl Institue Child Health Hum Dev Council (2023), 1–62.

[5] Harrison M Thorsten V Dudley D Parker C Koch M Hogue C. Stillbirth, inflammatory markers, and obesity: results from the stillbirth collaborative research network. Am J Perinat (2018) 35:1071–8. doi: 10.1055/s-0038-1639340 PMC6436964 29609190

[6] Robertson SA Care AS Moldenhauer LM. Regulatory T cells in embryo implantation and the immune response to pregnancy. J Clin Invest (2018) 128:4224–35. doi: 10.1172/jci122182 PMC6159994 30272581

[7] Bezemer RE Schoots MH Timmer A Scherjon SA Erwich JJHM van Goor H. Altered levels of decidual immune cell subsets in fetal growth restriction, stillbirth, and placental pathology. Front Immunol (2020) 11:1898. doi: 10.3389/fimmu.2020.01898 32973787 PMC7468421

[8] Aluvihare VR Kallikourdis M Betz AG. Regulatory T cells mediate maternal tolerance to the fetus. Nat Immunol (2004) 5:266–71. doi: 10.1038/ni1037 14758358

[9] Shima T Sasaki Y Itoh M Nakashima A Ishii N Sugamura K. Regulatory T cells are necessary for implantation and maintenance of early pregnancy but not late pregnancy in allogeneic mice. J Reprod Immunol (2010) 85:121–9. doi: 10.1016/j.jri.2010.02.006 20439117

[10] Rowe JH Ertelt JM Aguilera MN Farrar MA Way SS. Foxp3+ Regulatory T cell expansion required for sustaining pregnancy compromises host defense against prenatal bacterial pathogens. Cell Host Microbe (2011) 10:54–64. doi: 10.1016/j.chom.2011.06.005 21767812 PMC3140139

[11] Rowe JH Ertelt JM Xin L Way SS. Pregnancy imprints regulatory memory that sustains anergy to fetal antigen. Nature (2012) 490:102–6. doi: 10.1038/nature11462 PMC3465465 23023128

[12] Samstein RM Josefowicz SZ Arvey A Treuting PM Rudensky AY. Extrathymic generation of regulatory T cells in placental mammals mitigates maternal-fetal conflict. Cell (2012) 150:29–38. doi: 10.1016/j.cell.2012.05.031 22770213 PMC3422629

[13] Green ES Moldenhauer LM Groome HM Sharkey DJ Chin PY Care AS. Regulatory T cells are paramount effectors in progesterone regulation of embryo implantation and fetal growth. JCI Insight (2023) 8:e162995. doi: 10.1172/jci.insight.162995 37191999 PMC10393240

[14] Teles A Schumacher A Kühnle M-C Linzke N Thuere C Reichardt P. Control of uterine microenvironment by Foxp3+ Cells facilitates embryo implantation. Front Immunol (2013) 4:158. doi: 10.3389/fimmu.2013.00158 23801995 PMC3689029

[15] Kahn DA Baltimore D. Pregnancy induces a fetal antigen-specific maternal T regulatory cell response that contributes to tolerance. Proc Natl Acad Sci (2010) 107:9299–304. doi: 10.1073/pnas.1003909107 PMC2889122 20439708

[16] Tilburgs T Strominger JL. CD8+ Effector T cells at the fetal–maternal interface, balancing fetal tolerance and antiviral immunity. Am J Reprod Immunol (2013) 69:395–407. doi: 10.1111/aji.12094 23432707 PMC3711858

[17] Barton BM Xu R Wherry EJ Porrett PM. Pregnancy promotes tolerance to future offspring by programming selective dysfunction in long-lived maternal T cells. J Leukocyte Biol (2017) 101:975–87. doi: 10.1189/jlb.1a0316-135r PMC12040000 27810945

[18] Kinder JM Turner LH Stelzer IA Miller-Handley H Burg A Shao T-Y. CD8+ T cell functional exhaustion overrides pregnancy-induced fetal antigen alloimmunization. Cell Rep (2020) 31:107784. doi: 10.1016/j.celrep.2020.107784 32579916 PMC7383938

[19] Suah AN Tran D-KV Khiew SHW Andrade MS Pollard JM Jain D. Pregnancy-induced humoral sensitization overrides T cell tolerance to fetus-matched allografts in mice. J Clin Invest (2021) 131:e140715. doi: 10.1172/jci140715 33393512 PMC7773355

[20] Lewis EL Xu R Beltra J-C Ngiow SF Cohen J Telange R. NFAT-dependent and -independent exhaustion circuits program maternal CD8 T cell hypofunction in pregnancy. J Exp Med (2021) 219:e20201599. doi: 10.1084/jem.20201599 34882194 PMC8666877

[21] Woidacki K Meyer N Schumacher A Goldschmidt A Maurer M Zenclussen AC. Transfer of regulatory T cells into abortion-prone mice promotes the expansion of uterine mast cells and normalizes early pregnancy angiogenesis. Sci Rep (2015) 5:13938. doi: 10.1038/srep13938 26355667 PMC4565045

[22] Care AS Bourque SL Morton JS Hjartarson EP Robertson SA Davidge ST. Reduction in regulatory T cells in early pregnancy causes uterine artery dysfunction in mice. Hypertension (2018) 72:177–87. doi: 10.1161/hypertensionaha.118.10858 29785960

[23] Schepper SD Verheijden S Aguilera-Lizarraga J Viola MF Boesmans W Stakenborg N. Self-maintaining gut macrophages are essential for intestinal homeostasis. Cell (2018) 175:400–415.e13. doi: 10.1016/j.cell.2018.07.048 30173915

[24] Yamasaki K van Eeden SF. Lung macrophage phenotypes and functional responses: role in the pathogenesis of COPD. Int J Mol Sci (2018) 19:582. doi: 10.3390/ijms19020582 29462886 PMC5855804

[25] Braga FAV Kar G Berg M Carpaij OA Polanski K Simon LM. A cellular census of human lungs identifies novel cell states in health and in asthma. Nat Med (2019) 25:1153–63. doi: 10.1038/s41591-019-0468-5 31209336

[26] Lin D Yang L Wen L Lu H Chen Q Wang Z. Crosstalk between the oral microbiota, mucosal immunity, and the epithelial barrier regulates oral mucosal disease pathogenesis. Mucosal Immunol (2021) 14:1247–58. doi: 10.1038/s41385-021-00413-7 34040155

[27] Islam EA Shaik-Dasthagirisaheb Y Kaushic C Wetzler LM Gray-Owen SD. The reproductive cycle is a pathogenic determinant during gonococcal pelvic inflammatory disease in mice. Mucosal Immunol (2016) 9:1051–64. doi: 10.1038/mi.2015.122 PMC4915993 26693700

[28] Brooks RA Fleming GF Lastra RR Lee NK Moroney JW Son CH. Current recommendations and recent progress in endometrial cancer. CA Cancer J Clin (2019) 69:258–79. doi: 10.3322/caac.21561 31074865

[29] Li W Lin A Qi L Lv X Yan S Xue J. Immunotherapy: A promising novel endometriosis therapy. Front Immunol (2023) 14:1128301. doi: 10.3389/fimmu.2023.1128301 37138868 PMC10150018

[30] Hogg C Horne AW Greaves E. Endometriosis-associated macrophages: origin, phenotype, and function. Front Endocrinol (2020) 11:7. doi: 10.3389/fendo.2020.00007 PMC6989423 32038499

[31] O’Malley DM Bariani GM Cassier PA Marabelle A Hansen AR Acosta ADJ. Pembrolizumab in patients with microsatellite instability–high advanced endometrial cancer: results from the KEYNOTE-158 study. J Clin Oncol (2022) 40:752–61. doi: 10.1200/jco.21.01874 PMC8887941 34990208

[32] Erlebacher A. Immunology of the maternal-fetal interface. Annu Rev Immunol (2013) 31:387–411. doi: 10.1146/annurev-immunol-032712-100003 23298207

[33] Cappelletti M Presicce P Kallapur SG. Immunobiology of acute chorioamnionitis. Front Immunol (2020) 11:649. doi: 10.3389/fimmu.2020.00649 32373122 PMC7177011

[34] Pan D Liu Q Du L Yang Y Jiang G. Polarization disorder of decidual NK cells in unexplained recurrent spontaneous abortion revealed by single-cell transcriptome analysis. Reprod Biol Endocrin (2022) 20:108. doi: 10.1186/s12958-022-00980-9 PMC9327377 35897028

[35] Guerrero B Hassouneh F Delgado E Casado JG Tarazona R. Natural killer cells in recurrent miscarriage: An overview. J Reprod Immunol (2020) 142:103209. doi: 10.1016/j.jri.2020.103209 32992208

[36] Csabai T Pallinger E Kovacs AF Miko E Bognar Z Szekeres-Bartho J. Altered immune response and implantation failure in progesterone-induced blocking factor-deficient mice. Front Immunol (2020) 11:349. doi: 10.3389/fimmu.2020.00349 32218780 PMC7079574

[37] Shmeleva EV Colucci F. Maternal natural killer cells at the intersection between reproduction and mucosal immunity. Mucosal Immunol (2021) 14:991–1005. doi: 10.1038/s41385-020-00374-3 33903735 PMC8071844

[38] Moffett A Shreeve N. First do no harm: uterine natural killer (NK) cells in assisted reproduction. Hum Reprod (2015) 30:1519–25. doi: 10.1093/humrep/dev098 PMC4472320 25954039

[39] Sheikhansari G Pourmoghadam Z Danaii S Mehdizadeh A Yousefi M. Etiology and management of recurrent implantation failure: a focus on Intra-uterine PBMC-therapy for RIF. J Reprod Immunol (2020) 139:103121. doi: 10.1016/j.jri.2020.103121 32240947

[40] Krop J van der Zwan A Ijsselsteijn ME Kapsenberg H Luk SJ Hendriks SH. Imaging mass cytometry reveals the prominent role of myeloid cells at the maternal-fetal interface. iScience (2022) 25:104648. doi: 10.1016/j.isci.2022.104648 35811852 PMC9257341

[41] Bao S Chen Z Qin D Xu H Deng X Zhang R. Single-cell profiling reveals mechanisms of uncontrolled inflammation and glycolysis in decidual stromal cell subtypes in recurrent miscarriage. Hum Reprod (2022) 38:57–74. doi: 10.1093/humrep/deac240 36355621

[42] Chen P Zhou L Chen J Lu Y Cao C Lv S. The immune atlas of human deciduas with unexplained recurrent pregnancy loss. Front Immunol (2021) 12:689019. doi: 10.3389/fimmu.2021.689019 34168655 PMC8218877

[43] Wang F Jia W Fan M Shao X Li Z Liu Y. Single-cell immune landscape of human recurrent miscarriage. Genom Proteom Bioinform (2021) 19:208–22. doi: 10.1016/j.gpb.2020.11.002 PMC8602400 33482359

[44] Zenclussen AC Gerlof K Zenclussen ML Sollwedel A Bertoja AZ Ritter T. Abnormal T-cell reactivity against paternal antigens in spontaneous abortion adoptive transfer of pregnancy-induced CD4+CD25+ T regulatory cells prevents fetal rejection in a murine abortion model. Am J Pathol (2005) 166:811–22. doi: 10.1016/s0002-9440(10)62302-4 PMC1602357 15743793

[45] Bonney EA Brown SA. To drive or be driven: the path of a mouse model of recurrent pregnancy loss. Reproduction (2014) 147:R153–67. doi: 10.1530/rep-13-0583 PMC4316810 24472815

[46] Blois S Soto CDA Olmos S Chuluyan E Gentile T Arck PC. Therapy with dendritic cells influences the spontaneous resorption rate in the CBA/J × DBA/2J mouse model. Am J Reprod Immunol (2004) 51:40–8. doi: 10.1046/j.8755-8920.2003.00120.x 14725565

[47] Xu W Xiao Z Wang X Huang Y. IL-17 induces fetal loss in a CBA/J×BALB/c mouse model, and an anti-IL-17 antibody prevents fetal loss in a CBA/J ×DBA/2 mouse model. Am J Reprod Immunol (2016) 75:51–8. doi: 10.1111/aji.12437 26474535

[48] Clark DA Manuel J Lee L Chaouat G Gorczynski RM Levy GA. Ecology of danger-dependent cytokine-boosted spontaneous abortion in the CBA × DBA/2 mouse model. I. Synergistic effect of LPS and (TNF-α + IFN-γ) on pregnancy loss. Am J Reprod Immunol (2004) 52:370–8. doi: 10.1111/j.1600-0897.2004.00237.x 15663602

[49] Zhang J Wei H Wu D Tian Z. Toll-like receptor 3 agonist induces impairment of uterine vascular remodeling and fetal losses in CBA×DBA/2 mice. J Reprod Immunol (2007) 74:61–7. doi: 10.1016/j.jri.2006.10.005 17196665

[50] Wang S Chen C Li M Qian J Sun F Li Y. Blockade of CTLA-4 and Tim-3 pathways induces fetal loss with altered cytokine profiles by decidual CD4+T cells. Cell Death Dis (2019) 10:15. doi: 10.1038/s41419-018-1251-0 30622243 PMC6325160

[51] Li G Yang L Li D Zhang J Du L Xia L. Effects of combined treatment with PD-L1 Ig and CD40L mAb on immune tolerance in the CBA/J × DBA/2 mouse model. Mol Med Rep (2020) 21:1789–98. doi: 10.3892/mmr.2020.10977 PMC7057827 32319625

[52] Wang W-J Liu F-J Xin-Liu Hao C-F Bao H-C Qu Q-L. Adoptive transfer of pregnancy-induced CD4+CD25+ regulatory T cells reverses the increase in abortion rate caused by interleukin 17 in the CBA/J×BALB/c mouse model. Hum Reprod (2014) 29:946–52. doi: 10.1093/humrep/deu014 24556316

[53] Idali F Rezaii-nia S Golshahi H Fatemi R Naderi MM Goli LB. Adoptive cell therapy with induced regulatory T cells normalises the abortion rate in abortion-prone mice. Reprod Fertil Dev (2020) 33:220–228. doi: 10.1071/rd20063 33317684

[54] Wang J Yang J Yan Y Zhu Z Mu Y Wang X. Effect of adoptive transfer of CD4+CD25+Foxp3+ Treg induced by trichostatin A on the prevention of spontaneous abortion. J Reprod Immunol (2019) 131:30–5. doi: 10.1016/j.jri.2018.12.002 30634133

[55] Yin Y Han X Shi Q Zhao Y He Y. Adoptive transfer of CD4+CD25+ regulatory T cells for prevention and treatment of spontaneous abortion. Eur J Obstet Gyn R B (2012) 161:177–81. doi: 10.1016/j.ejogrb.2011.12.023 22261465

[56] Groner B Hynes NE. Number and location of mouse mammary tumor virus proviral DNA in mouse DNA of normal tissue and of mammary tumors. J Virol (1980) 33:1013–25. doi: 10.1128/jvi.33.3.1013-1025.1980 PMC288635 6245257

[57] Tiemessen MM Jagger AL Evans HG van Herwijnen MJC John S Taams LS. CD4+CD25+Foxp3+ regulatory T cells induce alternative activation of human monocytes/macrophages. Proc Natl Acad Sci (2007) 104:19446–51. doi: 10.1073/pnas.0706832104 PMC2148309 18042719

[58] Meng Y-H Zhou W-J Jin L-P Liu L-B Chang K-K Mei J. RANKL-mediated harmonious dialogue between fetus and mother guarantees smooth gestation by inducing decidual M2 macrophage polarization. Cell Death Dis (2017) 8:e3105–5. doi: 10.1038/cddis.2017.505 PMC5682671 29022922

[59] Zhou Y Zhou B Pache L Chang M Khodabakhshi AH Tanaseichuk O. Metascape provides a biologist-oriented resource for the analysis of systems-level datasets. Nat Commun (2019) 10:1523. doi: 10.1038/s41467-019-09234-6 30944313 PMC6447622

[60] Weinberger T Esfandyari D Messerer D Percin G Schleifer C Thaler R. Ontogeny of arterial macrophages defines their functions in homeostasis and inflammation. Nat Commun (2020) 11:4549. doi: 10.1038/s41467-020-18287-x 32917889 PMC7486394

[61] Mildner A Schönheit J Giladi A David E Lara-Astiaso D Lorenzo-Vivas E. Genomic characterization of murine monocytes reveals C/EBPβ Transcription factor dependence of Ly6C– cells. Immunity (2017) 46:849–862.e7. doi: 10.1016/j.immuni.2017.04.018 28514690

[62] Keren-Shaul H Spinrad A Weiner A Matcovitch-Natan O Dvir-Szternfeld R Ulland TK. A unique microglia type associated with restricting development of Alzheimer’s disease. Cell (2017) 169:1276–1290.e17. doi: 10.1016/j.cell.2017.05.018 28602351

[63] Cochain C Vafadarnejad E Arampatzi P Pelisek J Winkels H Ley K. Single-cell RNA-seq reveals the transcriptional landscape and heterogeneity of aortic macrophages in murine atherosclerosis. Circ Res (2018) 122:1661–74. doi: 10.1161/circresaha.117.312509 29545365

[64] Burl RB Ramseyer VD Rondini EA Pique-Regi R Lee Y-H Granneman JG. Deconstructing adipogenesis induced by β3-adrenergic receptor activation with single-cell expression profiling. Cell Metab (2018) 28:300–309.e4. doi: 10.1016/j.cmet.2018.05.025 29937373 PMC6082711

[65] Jaitin DA Adlung L Thaiss CA Weiner A Li B Descamps H. Lipid-associated macrophages control metabolic homeostasis in a Trem2-dependent manner. Cell (2019) 178:686–698.e14. doi: 10.1016/j.cell.2019.05.054 31257031 PMC7068689

[66] Xiong X Kuang H Ansari S Liu T Gong J Wang S. Landscape of intercellular crosstalk in healthy and NASH liver revealed by single-cell secretome gene analysis. Mol Cell (2019) 75:644–660.e5. doi: 10.1016/j.molcel.2019.07.028 31398325 PMC7262680

[67] Wang ECE Dai Z Ferrante AW Drake CG Christiano AM. A subset of TREM2+ Dermal macrophages secretes oncostatin M to maintain hair follicle stem cell quiescence and inhibit hair growth. Cell Stem Cell (2019) 24:654–669.e6. doi: 10.1016/j.stem.2019.01.011 30930146

[68] Zilionis R Engblom C Pfirschke C Savova V Zemmour D Saatcioglu HD. Single-cell transcriptomics of human and mouse lung cancers reveals conserved myeloid populations across individuals and species. Immunity (2019) 50:1317–1334.e10. doi: 10.1016/j.immuni.2019.03.009 30979687 PMC6620049

[69] Zhang L Li Z Skrzypczynska KM Fang Q Zhang W O’Brien SA. Single-cell analyses inform mechanisms of myeloid-targeted therapies in colon cancer. Cell (2020) 181:442–459.e29. doi: 10.1016/j.cell.2020.03.048 32302573

[70] Katzenelenbogen Y Sheban F Yalin A Yofe I Svetlichnyy D Jaitin DA. Coupled scRNA-Seq and intracellular protein activity reveal an immunosuppressive role of TREM2 in cancer. Cell (2020) 182:872–885.e19. doi: 10.1016/j.cell.2020.06.032 32783915

[71] Harasymowicz NS Rashidi N Savadipour A Wu C Tang R Bramley J. Single-cell RNA sequencing reveals the induction of novel myeloid and myeloid-associated cell populations in visceral fat with long-term obesity. FASEB J (2021) 35:e21417. doi: 10.1096/fj.202001970r 33566380 PMC8743141

[72] Antunes ARP Scheyltjens I Lodi F Messiaen J Antoranz A Duerinck J. Single-cell profiling of myeloid cells in glioblastoma across species and disease stage reveals macrophage competition and specialization. Nat Neurosci (2021) 24:595–610. doi: 10.1038/s41593-020-00789-y 33782623

[73] Vento-Tormo R Efremova M Botting RA Turco MY Vento-Tormo M Meyer KB. Single-cell reconstruction of the early maternal–fetal interface in humans. Nature (2018) 563:347–53. doi: 10.1038/s41586-018-0698-6 PMC7612850 30429548

[74] Sureshchandra S Zulu MZ Doratt BM Jankeel A Tifrea D Edwards R. Single-cell RNA sequencing reveals immunological rewiring at the maternal-fetal interface following asymptomatic/mild SARS-CoV-2 infection. Cell Rep (2022) 39:110938. doi: 10.1016/j.celrep.2022.110938 35662411 PMC9130636

[75] Bertoja AZ Zenclussen ML Casalis PA Sollwedel A Schumacher A Woiciechowsky C. Anti-P- and E-selectin therapy prevents abortion in the CBA/J × DBA/2J combination by blocking the migration of Th1 lymphocytes into the foetal–maternal interface. Cell Immunol (2005) 238:97–102. doi: 10.1016/j.cellimm.2006.02.002 16579979

[76] Dixon ME Chien EK Osol G Callas PW Bonney EA. Failure of decidual arteriolar remodeling in the CBA/J × DBA/2 murine model of recurrent pregnancy loss is linked to increased expression of tissue inhibitor of metalloproteinase 2 (TIMP-2). Am J Obstet Gynecol (2006) 194:113–9. doi: 10.1016/j.ajog.2005.06.063 16389019

[77] He M Jiang M Zhou Y Li F Yang M Fan Y. Impaired Gal-9 dysregulates the PBMC-induced Th1/Th2 imbalance in abortion-prone matings. J Immunol Res (2018) 2018:1–9. doi: 10.1155/2018/9517842 PMC5831994 29651447

[78] Hosseini MS Ali-Hassanzadeh M Nadimi E Karbalay-Doust S Noorafshan A Gharesi-Fard B. Stereological study of the placental structure in abortion-prone mice model (CBA/J×DBA/2J). Ann Anat Anatomischer Anzeiger (2020) 230:151508. doi: 10.1016/j.aanat.2020.151508 32173562

[79] Cheng H Huang Y Huang G Chen Z Tang J Pan L. Effect of the IDO gene on pregnancy in mice with recurrent pregnancy loss. Reprod Sci (2021) 28:52–9. doi: 10.1007/s43032-020-00264-w 32725590

[80] Page JM Christiansen-Lindquist L Thorsten V Parker CB Reddy UM Dudley DJ. Diagnostic tests for evaluation of stillbirth. Obstet Gynecol (2017) 129:699–706. doi: 10.1097/aog.0000000000001937 28333795 PMC12935483

[81] Baines MG Duclos AJ Antecka E Haddad EK. Decidual infiltration and activation of macrophages leads to early embryo loss. Am J Reprod Immunol (1997) 37:471–7. doi: 10.1111/j.1600-0897.1997.tb00262.x 9228304

[82] Gustafsson C Mjösberg J Matussek A Geffers R Matthiesen L Berg G. Gene expression profiling of human decidual macrophages: evidence for immunosuppressive phenotype. PloS One (2008) 3:e2078. doi: 10.1371/journal.pone.0002078 18446208 PMC2323105

[83] Zhang Y Ma L Hu X Ji J Mor G Liao A. The role of the PD-1/PD-L1 axis in macrophage differentiation and function during pregnancy. Hum Reprod (2018) 34:25–36. doi: 10.1093/humrep/dey347 30500923

[84] Proto JD Doran AC Gusarova G Yurdagul A Sozen E Subramanian M. Regulatory T cells promote macrophage efferocytosis during inflammation resolution. Immunity (2018) 49:666–677.e6. doi: 10.1016/j.immuni.2018.07.015 30291029 PMC6192849

[85] Batlle E Massagué J. Transforming growth factor-β Signaling in immunity and cancer. Immunity (2019) 50:924–40. doi: 10.1016/j.immuni.2019.03.024 PMC7507121 30995507

[86] Sharp AN Heazell AEP Crocker IP Mor G. Placental apoptosis in health and disease. Am J Reprod Immunol (2010) 64:159–69. doi: 10.1111/j.1600-0897.2010.00837.x PMC3025811 20367628

[87] Madhukaran SP Kishore U Jamil K Teo BHD Choolani M Lu J. Transcriptional factor PU.1 regulates decidual C1q expression in early pregnancy in human. Front Immunol (2015) 6:53. doi: 10.3389/fimmu.2015.00053 25762996 PMC4329821

[88] Deczkowska A Weiner A Amit I. The physiology, pathology, and potential therapeutic applications of the TREM2 signaling pathway. Cell (2020) 181:1207–17. doi: 10.1016/j.cell.2020.05.003 32531244

[89] Hsieh CL Koike M Spusta SC Niemi EC Yenari M Nakamura MC. A role for TREM2 ligands in the phagocytosis of apoptotic neuronal cells by microglia. J Neurochem (2009) 109:1144–56. doi: 10.1111/j.1471-4159.2009.06042.x PMC3087597 19302484

[90] Singh J Ahmed A Girardi G. Role of complement component C1q in the onset of preeclampsia in mice. Hypertension (2011) 58:716–24. doi: 10.1161/hypertensionaha.111.175919 21859968

[91] Agostinis C Bulla R Tripodo C Gismondi A Stabile H Bossi F. An alternative role of C1q in cell migration and tissue remodeling: contribution to trophoblast invasion and placental development. J Immunol (2010) 185:4420–9. doi: 10.4049/jimmunol.0903215 20810993

[92] Belmonte B Mangogna A Gulino A Cancila V Morello G Agostinis C. Distinct roles of classical and lectin pathways of complement in preeclamptic placentae. Front Immunol (2022) 13:882298. doi: 10.3389/fimmu.2022.882298 35711467 PMC9197446

[93] Agostinis C Tedesco F Bulla R. Alternative functions of the complement protein C1q at embryo implantation site. J Reprod Immunol (2017) 119:74–80. doi: 10.1016/j.jri.2016.09.001 27687635

[94] Madhukaran SP Kishore U Jamil K Choolani M Lu J. Decidual expression and localization of human surfactant protein SP-A and SP-D, and complement protein C1q. Mol Immunol (2015) 66:197–207. doi: 10.1016/j.molimm.2015.03.001 25829244

[95] Agostinis C Stampalija T Tannetta D Loganes C Brumatti LV Seta FD. Complement component C1q as potential diagnostic but not predictive marker of preeclampsia. Am J Reprod Immunol (2016) 76:475–81. doi: 10.1111/aji.12586 27666323

[96] Woo J-H Yang Y-I Ahn J-H Choi YS Choi J-H. Interleukin 6 secretion from alternatively activated macrophages promotes the migration of endometriotic epithelial cells. Biol Reprod (2017) 97:660–70. doi: 10.1093/biolre/iox118 29036448

[97] Mor G Aldo P Alvero AB. The unique immunological and microbial aspects of pregnancy. Nat Rev Immunol (2017) 17:469–82. doi: 10.1038/nri.2017.64 28627518

[98] Binnewies M Pollack JL Rudolph J Dash S Abushawish M Lee T. Targeting TREM2 on tumor-associated macrophages enhances immunotherapy. Cell Rep (2021) 37:109844. doi: 10.1016/j.celrep.2021.109844 34686340

[99] Ruganzu JB Zheng Q Wu X He Y Peng X Jin H. TREM2 overexpression rescues cognitive deficits in APP/PS1 transgenic mice by reducing neuroinflammation *via* the JAK/STAT/SOCS signaling pathway. Exp Neurol (2021) 336:113506. doi: 10.1016/j.expneurol.2020.113506 33065077

[100] Ayyubova G. TREM2 signalling as a multifaceted player in brain homoeostasis and a potential target for Alzheimer’s disease treatment. Eur J Neurosci (2023) 57:718–33. doi: 10.1111/ejn.15914 36637116

[101] Price BR Sudduth TL Weekman EM Johnson S Hawthorne D Woolums A. Therapeutic Trem2 activation ameliorates amyloid-beta deposition and improves cognition in the 5XFAD model of amyloid deposition. J Neuroinflamm (2020) 17:238. doi: 10.1186/s12974-020-01915-0 PMC7427742 32795308

[102] Varghese PM Kishore U Rajkumari R. Human C1q regulates influenza A virus infection and inflammatory response *via* its globular domain. Int J Mol Sci (2022) 23:3045. doi: 10.3390/ijms23063045 35328462 PMC8949502

[103] Li NL Birmingham DJ Rovin BH. Expanding the role of complement therapies: the case for lupus nephritis. J Clin Med (2021) 10:626. doi: 10.3390/jcm10040626 33562189 PMC7915321

[104] Lansita JA Mease KM Qiu H Yednock T Sankaranarayanan S Kramer S. Nonclinical development of ANX005: A humanized anti-C1q antibody for treatment of autoimmune and neurodegenerative diseases. Int J Toxicol (2017) 36:449–62. doi: 10.1177/1091581817740873 29202623

[105] Espericueta V Manughian-Peter AO Bally I Thielens NM Fraser DA. Recombinant C1q variants modulate macrophage responses but do not activate the classical complement pathway. Mol Immunol (2020) 117:65–72. doi: 10.1016/j.molimm.2019.10.008 31739194 PMC6931381

[106] Harden PN Game DS Sawitzki B der Net JBV Hester J Bushell A. Feasibility, long-term safety, and immune monitoring of regulatory T cell therapy in living donor kidney transplant recipients. Am J Transplant (2021) 21:1603–11. doi: 10.1111/ajt.16395 PMC7613119 33171020

[107] Herold KC Bundy BN Long SA Bluestone JA DiMeglio LA Dufort MJ. An anti-CD3 antibody, teplizumab, in relatives at risk for type 1 diabetes. N Engl J Med (2019) 381:603–13. doi: 10.1056/nejmoa1902226 PMC6776880 31180194

[108] Seelig E Howlett J Porter L Truman L Heywood J Kennet J. The DILfrequency study is an adaptive trial to identify optimal IL-2 dosing in patients with type 1 diabetes. JCI Insight (2018) 3:e99306. doi: 10.1172/jci.insight.99306 30282826 PMC6237447

[109] Bluestone JA Buckner JH Fitch M Gitelman SE Gupta S Hellerstein MK. Type 1 diabetes immunotherapy using polyclonal regulatory T cells. Sci Transl Med (2015) 7:315ra189. doi: 10.1126/scitranslmed.aad4134 PMC4729454 26606968

[110] Cai S Dai S Lin R Huang C Zeng Y Diao L. The effectiveness and safety of intrauterine infusion of autologous regulatory T cells (Tregs) in patients with recurrent pregnancy loss and low levels of endometrial FoxP3+ cells: A retrospective cohort study. Am J Reprod Immunol (2023) 90:e13735. doi: 10.1111/aji.13735 37491931

[111] Gomez-Lopez N Arenas-Hernandez M Romero R Miller D Garcia-Flores V Leng Y. Regulatory T cells play a role in a subset of idiopathic preterm labor/birth and adverse neonatal outcomes. Cell Rep (2020) 32:107874. doi: 10.1016/j.celrep.2020.107874 32640239 PMC7396155

[112] Robertson SA Green ES Care AS Moldenhauer LM Prins JR Hull ML. Therapeutic potential of regulatory T cells in preeclampsia—Opportunities and challenges. Front Immunol (2019) 10:478. doi: 10.3389/fimmu.2019.00478 30984163 PMC6448013

[113] Elovitz MA Wang Z Chien EK Rychlik DF Phillippe M. A new model for inflammation-induced preterm birth the role of platelet-activating factor and toll-like receptor-4. Am J Pathol (2003) 163:2103–11. doi: 10.1016/s0002-9440(10)63567-5 PMC1892431 14578208

[114] Burd I Brown A Gonzalez JM Chai J Elovitz MA. A mouse model of term chorioamnionitis: unraveling causes of adverse neurological outcomes. Reprod Sci (2011) 18:900–7. doi: 10.1177/1933719111398498 PMC3343123 21421895

[115] Chaouat G Meliani AA Martal J Raghupathy R Elliott JF Elliot J. IL-10 prevents naturally occurring fetal loss in the CBA x DBA/2 mating combination, and local defect in IL-10 production in this abortion-prone combination is corrected by in *vivo* injection of IFN-tau. J Immunol (1995) 154:4261–8. doi: 10.4049/jimmunol.154.9.4261 7722286

[116] Pique-Regi R Romero R Tarca AL Sendler ED Xu Y Garcia-Flores V. Single cell transcriptional signatures of the human placenta in term and preterm parturition. Elife (2019) 8:e52004. doi: 10.7554/elife.52004 31829938 PMC6949028

[117] Parker CB Hogue CJR Koch MA Willinger M Reddy UM Thorsten VR. Stillbirth Collaborative Research Network: design, methods and recruitment experience. Paediatr Perinat Ep (2011) 25:425–35. doi: 10.1111/j.1365-3016.2011.01218.x PMC3665402 21819424

[118] Lewis EL Sierra L-J Barila GO Brown AG Porrett PM Elovitz MA. Placental immune state shifts with gestational age. Am J Reprod Immunol (2018) 79:e12848. doi: 10.1111/aji.12848 29577513

[119] Stuart T Butler A Hoffman P Hafemeister C Papalexi E Mauck WM. Comprehensive integration of single-cell data. Cell (2019) 177:1888–1902.e21. doi: 10.1016/j.cell.2019.05.031 31178118 PMC6687398

[120] Subramanian A Tamayo P Mootha VK Mukherjee S Ebert BL Gillette MA. Gene set enrichment analysis: A knowledge-based approach for interpreting genome-wide expression profiles. P Natl Acad Sci (2005) 102:15545–50. doi: 10.1073/pnas.0506580102 PMC1239896 16199517

