# Supplementary material for: Regulatory T cell adoptive transfer alters uterine immune populations, increasing a novel MHC-IIlow macrophage associated with healthy pregnancy
Source: Front Immunol. 2023 Oct 13;14:1256453. doi: 10.3389/fimmu.2023.1256453 (PMC10611509; doi:10.3389/fimmu.2023.1256453)
Supplement: Supplementary file 1 [file DataSheet_1.pdf]

Supplemental Figures
Lewis et al., 2023.

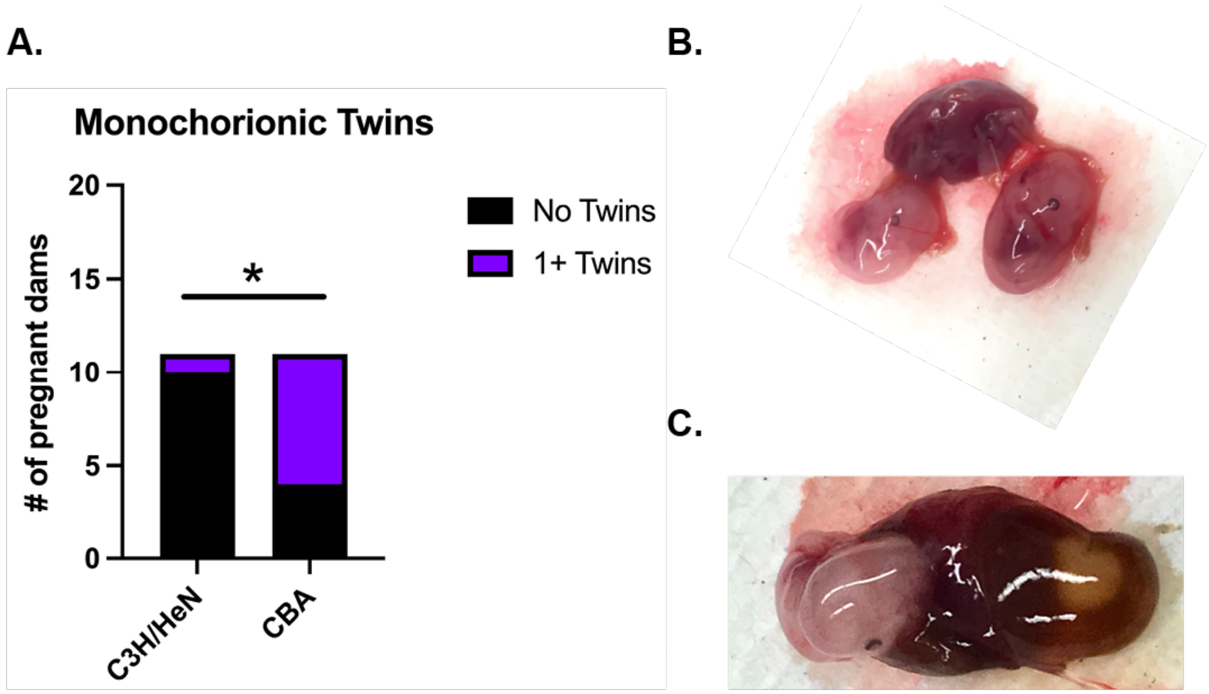

Figure S1: CBA pregnancies frequently contain monochorionic twins. C3H and CBA female mice were mated to DBA/2 males and sacrificed at E14 of pregnancy. (A) The presence of at least one monochorionic diamniotic twin was noted. (B) Representative photo of E14 twins from CBA pregnancy with amniotic sacs removed. (C) Representative photo of E14 twins from CBA pregnancy with one normal (left) and one demised (right) twin. Difference in twin frequency was assessed by Fisher's Exact Test. *:$p<0.05$.

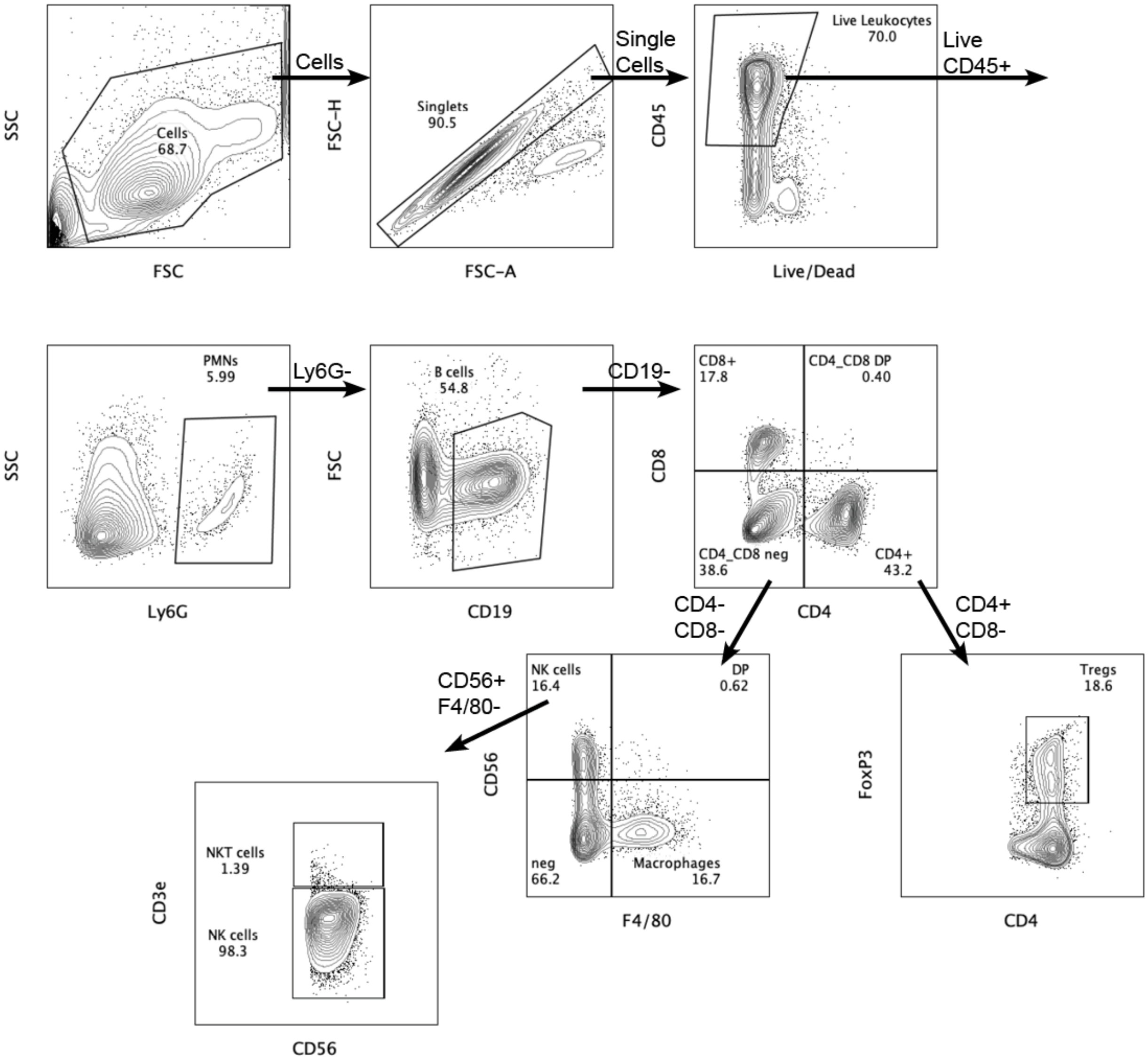

Figure S2: Flow cytometry gating scheme for gestational timecourse samples. The same gating scheme was used to analyze flow cytometry of tissues from both C3H and CBA pregnant mice at multiple gestational time points. Additional markers were added to further characterize macrophages (MHC-II, CD206, CD11b, CD11c, CD86, CD80, Trem2) in follow-up experiments, but the baseline gating is the same as above.

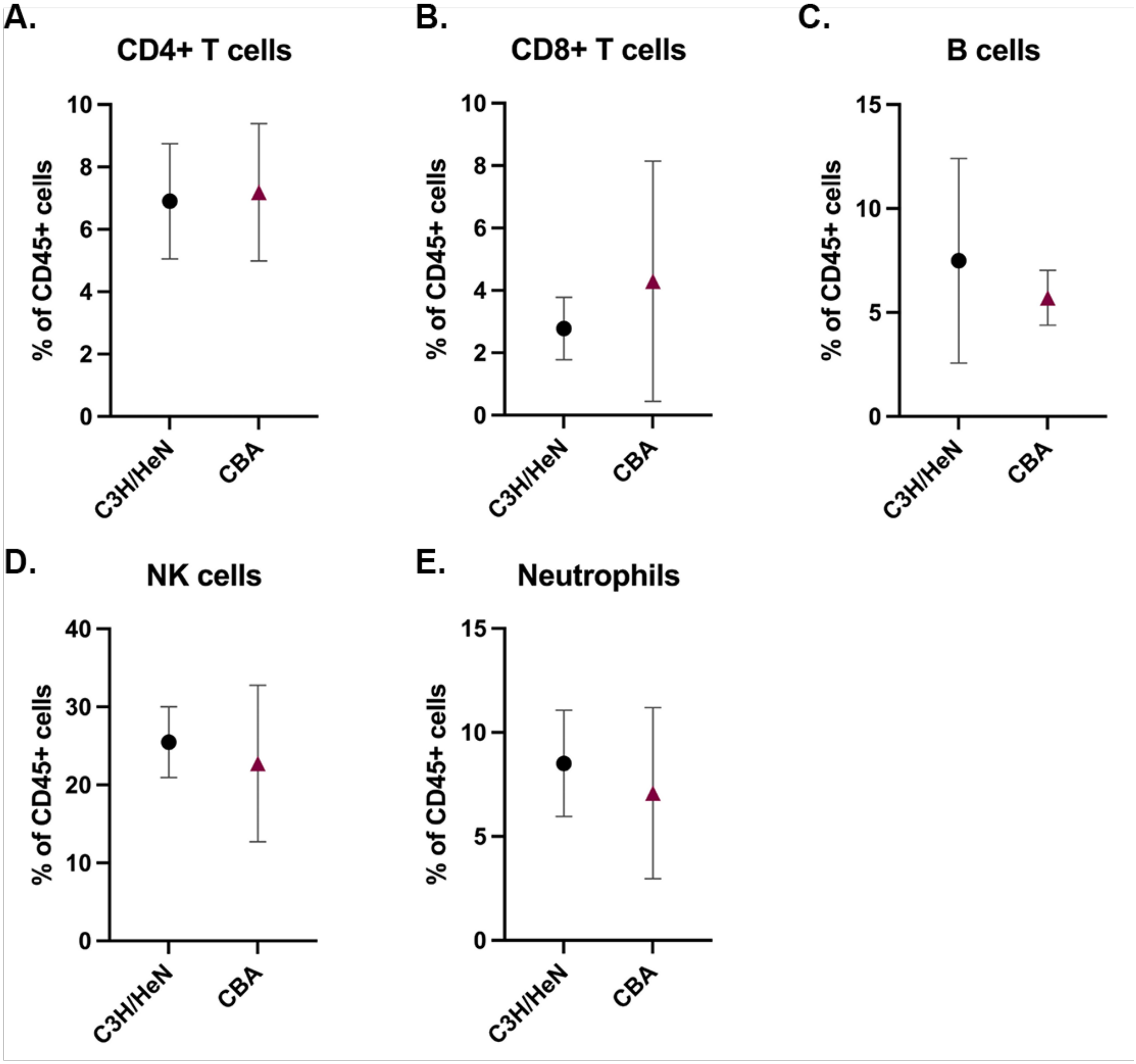

Figure S3: Uteri from virgin CBA and C3H/HeN mice show no difference in cellular immunologic composition. Healthy virgin CBA and C3H mice were sacrificed in estrus. Uterine immune cells were quantified by flow cytometry: (A) CD4+ T cells, (B) CD8+ T cells, (C) B cells, (D) NK cells, and (E) neutrophils. No difference between immune populations in CBA and C3H mice was found by unpaired t-test.

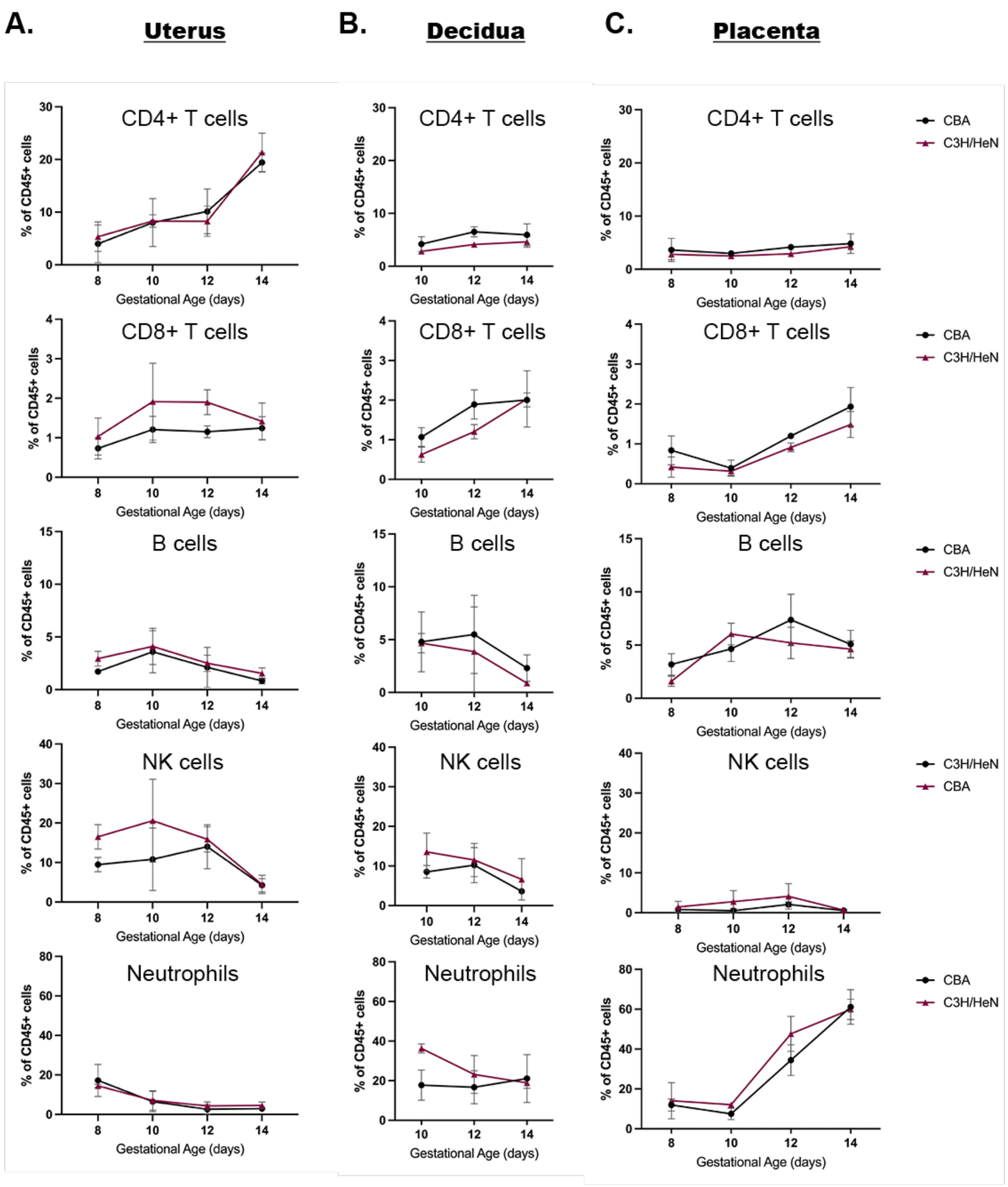

Figure S4: Immune populations at the maternal-fetal interface in CBA and C3H pregnancies. CBA and C3H female mice were mated to DBA/2 male mice. Mice were sacrificed every two days from E8 – E14 and immune cells in the (A) uterus, (B) decidua and (C) placenta were assessed by flow cytometry. At E8 there were too few decidua cells for analysis so (B)

decidual cells are only quantified from E10-E14. Quantitative differences between immune populations over gestation was assessed by two-way ANOVA, followed by Sidak's multiple comparison test comparing CBA to C3H rates of loss at the same gestational age if ANOVA was significant. No significant differences were detected by Sidak's test. N=4 mice/strain/gestational age.

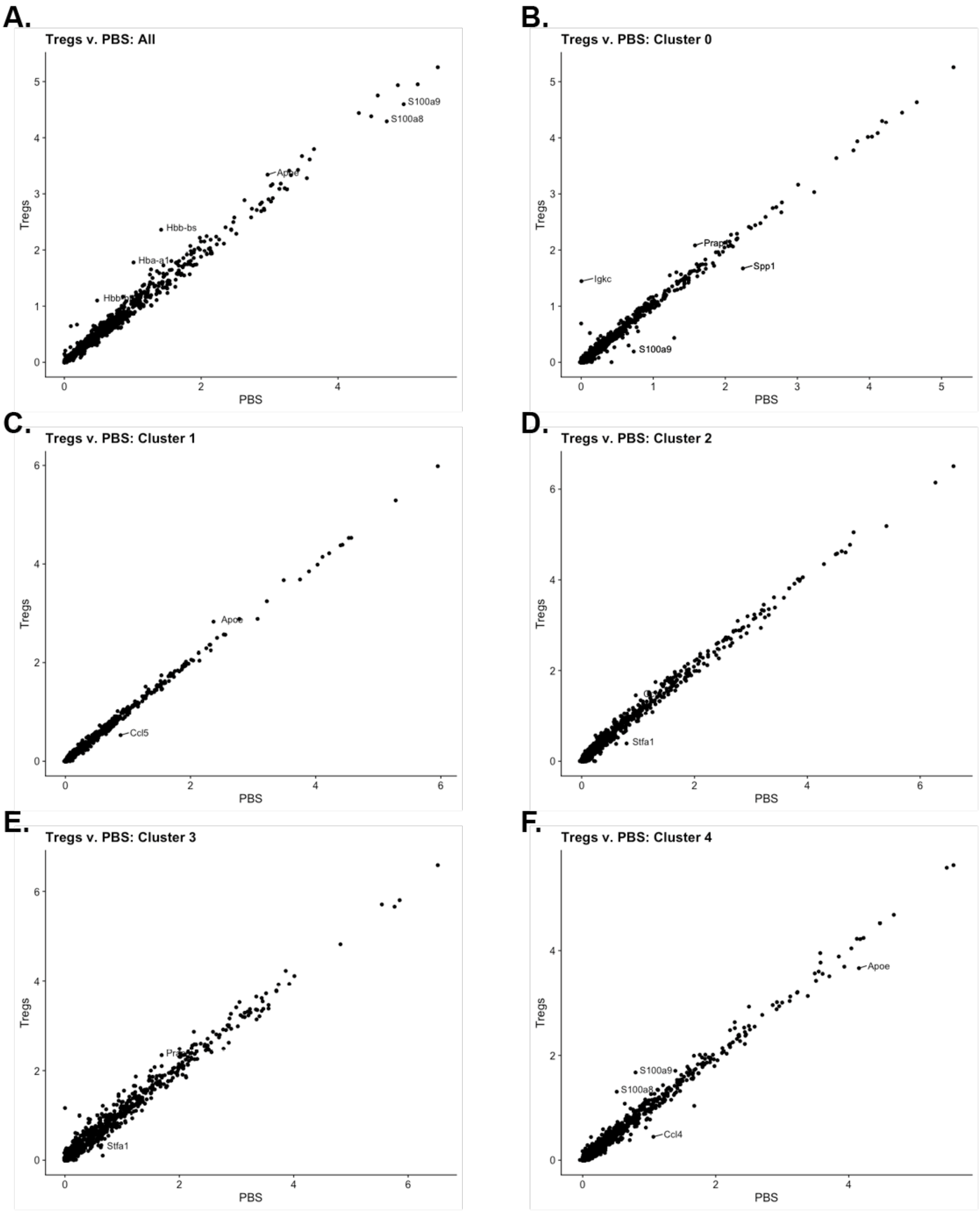

Figure S5: Differentially expressed genes between Treg-recipient and control mice. Single-cell RNA sequencing was performed on CD11b+ cells from the uteri of 3 Treg-recipient mice

and 3 PBS control mice. Overall, there were few differences in gene expression between (A) total Treg and PBS cells, or cells from Treg-recipient mice versus control mice within each cluster. Expression of genes from the (B-F) 5 largest clusters are shown.

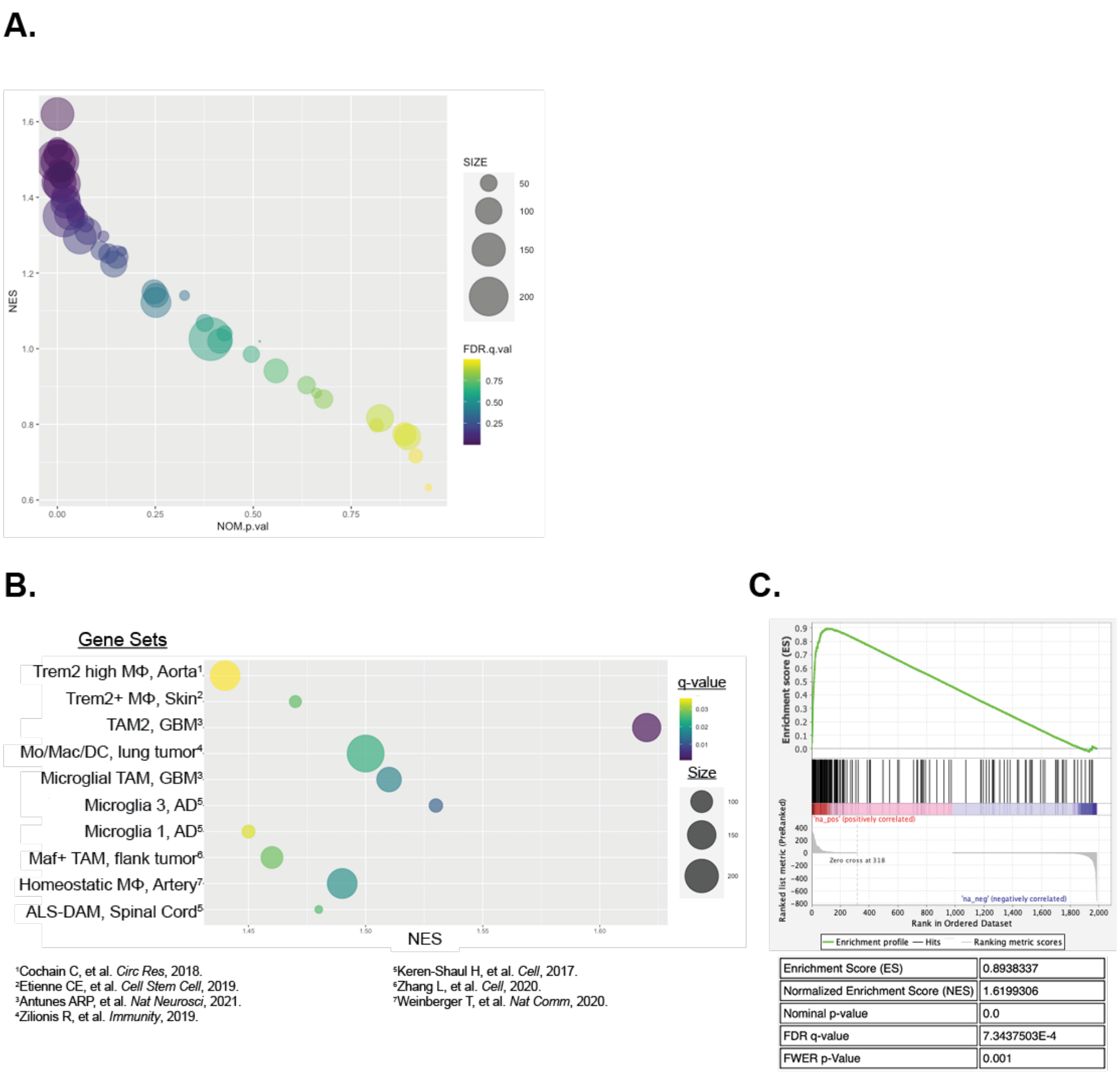

Figure S6: Upregulated gene lists in GSEA analysis. The gene cluster 0 gene set from our single-cell RNA-sequencing data was compared to a gene matrix of 123 gene lists. 47 of those gene lists were positively enriched in the cluster 0 gene set. (A) The normalized enrichment score (NES) of each of those gene sets is plotted against the nominal p-value (NOM.p.val), with the number of enriched genes plotted as dot size and the false discovery rate q-value (FDR.q.val) indicated by dot color. (B) 10 gene lists had enrichment scores with p-values < 0.01, shown in more detail with the x-axis as the NES, number of enriched genes plotted as dot size, and the FDR.q.value indicated by dot color. A gene list for tumor-associated macrophages (TAM) was

most significantly enriched in cluster 0 uterine macrophages (purple circle). (C) The gene set enrichment plot of the TAM gene list and cluster 0 macrophages is shown.

Supplemental tables are attached as excel documents.

Table S1: Differentially expressed genes between Treg-recipient and control mice for each scRNA-seq cluster.

Table S2: Defining genes for each scRNA-seq cluster.

Table S3: Core enriched genes between human decidual macrophages (MP2 from Pan, et al.) and mouse cluster 0 uterine macrophages.

Full Sequencing Data is available on the NIH Gene Expression Omnibus (GEO), accession number GSE244616.
